# Synthesis and molecular docking simulations of novel azepines based on quinazolinone moiety as prospective antimicrobial and antitumor hedgehog signaling inhibitors

**DOI:** 10.1038/s41598-024-53517-y

**Published:** 2024-02-12

**Authors:** Ahmed A. Noser, A. A. El-Barbary, Maha M. Salem, Hayam A. Abd El Salam, Mohamed shahien

**Affiliations:** 1 Organic Chemistry, Chemistry Department, Faculty of Science, Tanta University, Tanta, 31527 Egypt; 2 Biochemistry Division, Chemistry Department, Faculty of Science, Tanta University, Tanta, 31527 Egypt; 3 Green Chemistry Department, National Research Centre, Dokki, GizaCairo, 12622 Egypt

**Keywords:** Chemical biology, Chemistry

## Abstract

A series of novel azepine derivatives based on quinazolinone moiety was synthesized through the reaction of quinazolinone chalcones (**2a–d**) either with 2-amino aniline in acidic medium to give diazepines (**3a–d**) or with 2-aminophenol to offer oxazepine (**4a–d**). The structure of the synthesized compounds was confirmed via melting points, elemental analyses, and different spectroscopic techniques. Moreover, these newly compounds mode of action was investigated *in-silico* using molecular docking against the outer membrane protein A (OMPA), exo-1,3-beta-glucanase for their antimicrobial activity, and against Smoothened (SMO), transcription factor glioma-associated homology (SUFU/GLI-1), the main proteins of Hedgehog signaling pathway to inspect their anticancer potential. Our results showed that, diazepine (**3a**) and oxazepine (**4a**) offered the highest binding energy against the target OMPA/ exo-1,3-beta-glucanase proteins and exhibited the potent antimicrobial activities against *E. coli*, *P. aeruginosa*, *S. aureus*, *B. subtilis*, *C. Albicans* and *A. flavus*. As well, diazepine (**3a**) and oxazepine (**4a**) achieved the best results among the other compounds, in their binding energy against the target SMO, SUFU/GLI-1 proteins. The *in-vitro* cytotoxic study was done for them on panel of cancer cell lines HCT-116, HepG2, and MCF-7 and normal cell line WI-38. Conclusively, it was revealed that molecular docking *in-silico* simulations and the *in-vitro* experiments were agreed. As a result, our findings elucidated that diazepine (**3a**) and oxazepine (**4a**), have the potential to be used as antimicrobial agents and as possible cancer treatment medications.

## Introduction

The World Health Organization (WHO) estimates that antimicrobial resistance and cancer incidence remain the major concern diseases despite advances in preclinical and clinical research, due to a variety of heterogeneous risk factors including ethnicity, environmental exposure, gender, socioeconomic factors, genetic predisposition, location, and dietary habits^1^.

Antimicrobial resistance is influenced by outer membrane protein A (OMPA) and exo1,3 beta glucanases^2^. OMPA has a variety of roles in the pathophysiology of bacteria, including resistance, induction of host cell death, and adhesion to host cells. Clinically, overexpression of the OMPA gene is linked to the onset of pneumonia and bacteremia, as well as patient death^3^. Furthermore, β-1,3-glucanases is the primary skeletal polysaccharides of fungal cell walls that catalyzes the hydrolytic cleavage of the β-1,3-D-glycosidic linkages in β-1,3-glucans and it is the key enzyme in the lysis of phytopathogenic fungal cell walls during the pathogenicity, which appears to be the primary role for treatment^4^^.^

The Hedgehog (HH) cancer pathway is known to be involved in two cancer types: medulloblastoma, childhood cancer with an unfavorable prognosis, and basal cell carcinoma, which is the most frequent cancer in the Western world^5^. HH signaling has been found in almost 30% of human malignancies. When HH ligands (SHH, IHH, and DHH) bind to their receptor Patched 1 (PTCH) on the surface of target cells, HH signaling is canonically activated. Because Smoothened (SMO) is active. The downstream glioma-associated homologue (GLI) transcription factors GLI1 and GLI2 are activated via nuclear translocation, as ligand-bound PTCH loses its inhibitory influence on SMO^6^. The abnormal activation of this signal pathway is positively correlated with a poor prognosis because GLI target genes include elements important in cancer cell proliferation, survival, self-renewal, and invasiveness^7^. Thus, there is an urgent need to design and synthesize new compounds that act as SMO and GLI transcriptional direct inhibitors^8^.

**Figure 1:**
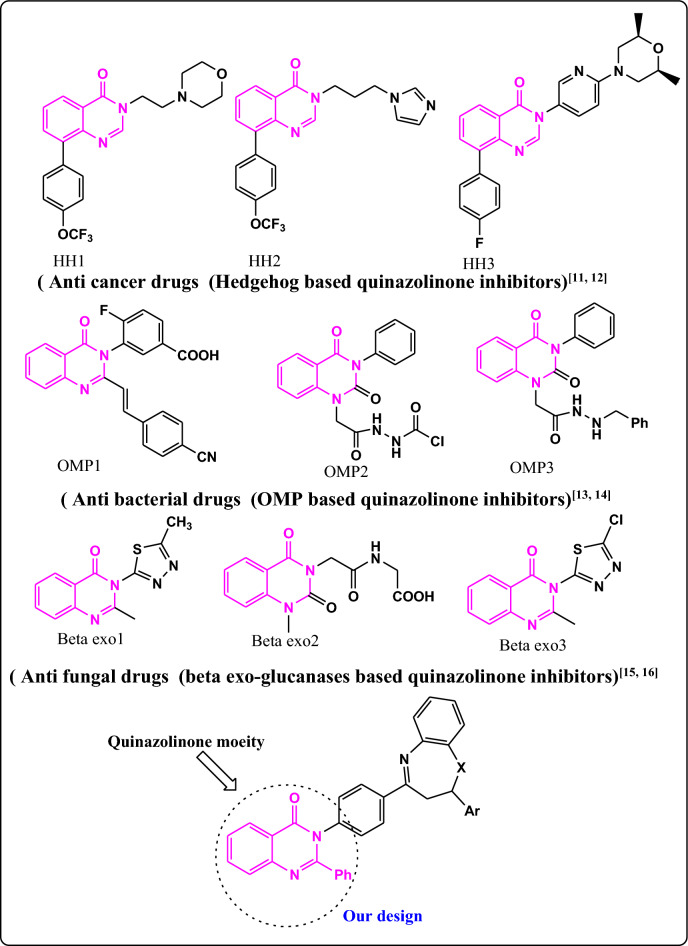
The rational design of the newly azepine derivatives based on quinazolinone moiety as antimicrobial and anticancer agents.

Nitrogen heterocycles, or quinazolinones, are one class of heterocyclic compounds with a broad range of uses. They are useful intermediates in medicinal chemistry and serve as structural components in physiologically active molecules. Because they can participate in a wide range of intermolecular interactions, including hydrogen bonds, metallic coordination bonds, van der Waals and hydrophobic forces, as well as different patterns of enzyme binding due to their wide range of ring sizes^9,10^. As illustrated in Fig. 1 the strong aromaticity of the ring and the presence of heteroatoms have been linked to the quinazolinone derivatives' diverse biological activities, which include anticancer activity via direct inhibiting of the HH signaling pathway^11,12^, antibacterial via inhibiting the target outer membrane protein (OMP)^13,14^, and antifungal candidates via suppressing the β-1,3-exoglucanases^15,16^. This strong aromaticity also contributes to the great *in- vivo* stability and low toxicity to higher vertebrates^17,18^. Further, Azepine-based compounds are receiving interest because seven membered heterocyclic azepines and their derivatives have important pharmacological and medical applications. Members of the benzodiazepine family are often employed as analgesics, anti-convulsant, anti-anxiolytics, anti-depressants, sedatives, and hypnotics^19,20^. Oxazepines also possess a range of other properties, including anti-fungal, anti-epileptic, anti-HIV, anti-histaminic, and anti-psychotic properties. Azepines are synthesized using a variety of methods in both conventional and environmental settings^21^.

Therefore, in the current study, a series of novel azepine derivatives based on quinazolinone moiety were designed, synthesized, characterized, and tested *in-silico* against the bacterial outer membrane protein A and the fungal- exo-1,3-beta-glucanase target proteins. Afterword the *in-silico* results were confirmed by examining their antimicrobial impact on several pathogenic strains. Additionally, to investigate the ability of these azepine derivatives in inhibiting the Hedgehog signaling pathway and elucidate their anticancer potential, molecular docking and *in-vitro* cytotoxic experiments were also carried out.

## Experimental section

### Chemistry

#### Chemicals and instrumentation

All chemicals and instrumentation are described in the supplementary file.

#### General procedure for the synthesis of chalcones (2a–d)

Equimolar amounts (10 mmol) of 3-(4-acetylphenyl)-2-phenylquinazolin-4(3H)-one (**1**) and different aromatic aldehydes were dissolved in ethanol (15 mL). Sodium hydroxide (0.08 g, 2 mmol) was added and stirred for 24 h. The reaction mixture was poured in to crushed ice, filtered, washed, and dried.

##### 2-phenyl-3-(4-((2E,4E)-5-phenylpenta-2,4-dienoyl) phenyl) quinazolin-4(3H)-one (2a)

Yield 70%; mp 148–150 °C; ^1^H NMR (400 MHz, DMSO d_6_) δ (ppm): 7.02–8.81 (m, 18H, Ar–H), 6.42 (d, 1H, CH-Ar), 7.10 (t, 1H, CH-CH-Ar), 7.14 (d, 1H, CH-C = O), 8.06 (t, 1H, CH-CH-C = O); ^13^C NMR (101 MHz, DMSO d_6_) δ (ppm): 186.52, 170.10, 164.41, 141.51, 135.21, 134.73, 132.81 131.76, 129.3, 127.73, 127.56, 123.61, 120.38, 117.11; IR (KBr) ν: 1680 (C=O), 1570 (C=N); Anal. Calcd for C_31_H_22_N_2_O_2_ (454.17): C, 81.92%; H, 4.88%; N, 6.16%. Found: C, 81.62%; H, 4.68%; N, 6.08%.

##### 3-(4-(3-(furan-3-yl)acryloyl)phenyl)-2-phenylquinazolin-4(3H)-one (2b)

Yield 82%; mp 170–172 °C; ^1^H NMR (400 MHz, DMSO d_6_) δ (ppm): 7.00–8.60 (m, 16H, Ar–H), 7.61 (d,1H, CH-CO), 8.02 (d,1H,CH-furan); ^13^C NMR (101 MHz, DMSO d_6_) δ (ppm): 182.12, 170.54, 165.23, 141.67, 135.10, 134.83, 132.69, 131.81, 129.61 127.63, 123.52, 120.53, 117.05; IR (KBr) ν: 1660 (C = O), 1575 (C = N); Anal. Calcd for C_27_H_18_N_2_O_3_ (418.45): C, 77.50%; H, 4.34%; N, 6.69%. Found: C, 77.32%; H, 4.22%; N, 6.47%.

##### 3-(4-cinnamoylphenyl)-2-phenylquinazolin-4(3H)-one (2c)

The structure of **2c** was confirmed as described earlier by Saravanan^22^.

##### The 3-(4-(3-(4-chlorophenyl) acryloyl) phenyl)-2-phenylquinazolin-4(3H)-one (2d)

The structure of **2d** was confirmed as described earlier^22^.

#### General procedure for the synthesis of azepines (3, 4)

Equimolar amount (1.0 mmol) of ethanolic solution of 2-amino aniline or 2-aminophenol and chalcone (**2a–d)** in glacial acetic acid (5.0 mL) were refluxed for 6–8 h. The reaction progress was monitored via TLC. The desired product was filtered off and dried under a vacuum.

##### 2-phenyl-3-(4-(2-styryl-2,3-dihydro-1H-benzo[b][1,4]diazepin-4-yl)phenyl)quinazolin-4(3H)-one (3a)

Yield 78%; mp 115–117 °C; ^1^H NMR (400 MHz, DMS*O*-d_6_) δ (ppm): 10.65 (s, 1H, NH), 7.10–8.22 (m, 22H, Ar–H), 3.35 (m, 1H, CH-NH), 2.18 (d 2H, CH_2_), 6.45 (dd, 1H, CH = CH-Ph), 6.70 (d,1H, CH = CH-Ph); ^13^C NMR (101 MHz, DMSO-d_6_) δ (ppm): 164.40, 159.30, 156.83, 146.80, 136.80, 129.43, 129.10, 128.50, 127.50, 116.80, 58.20, 32.90; IR (KBr) ν: 3620 (NH), 1730 (C=O), 1611 (C=N), 1251 (C–N) ; Anal. Calcd for C_37_H_28_N_4_O (544.23): C, 81.59%; H, 5.18%; N, 10.29%. Found: C, 81.38%; H, 5.08%; N, 10.05%.

##### 3-(4-(2-(furan-3-yl)-2,3-dihydro-1H-benzo[b][1,4]diazepin-4-yl)phenyl)-2-phenylquinazolin-4(3H)-one (3b)

Yield 74%; mp 150–152 °C; ^1^H NMR (400 MHz, DMSO-d_6_) δ (ppm): 11.72 (s, 1H, NH), 7.20–8.70 (m, 20H, Ar–H), 4.32 (t, 1H, CH–NH), 2.05 (d, 2H, CH_2_); ^13^C NMR (101 MHz, DMSO-d_6_) δ (ppm): 171.10, 168.22, 162.13, 141.90, 135.20, 135.00, 132.10, 131.50, 129.60 127.30, 123.13, 120.22, 116.50, 61.68, 34.40; IR (KBr) ν: 3300 (NH), 1683 (C=O), 1537 (C=N), 1231 (C–N); Anal. Calcd for C_33_H_24_N_4_O_2_ (508.19): C, 77.93%; H, 4.76%; N, 11.02%. Found: C, 77.76%; H, 4.48%; N, 10.84%.

##### 2-phenyl-3-(4-(2-phenyl-2,3-dihydro-1H-benzo[b][1,4]diazepin-4-yl)phenyl)quinazolin-4(3H)-one (3c)

Yield 75%; mp 170–172 °C; ^1^H NMR (400 MHz, DMSO-d_6_) δ (ppm): 11.40 (s, 1H, NH), 7.00–8.60 (m, 22H, Ar–H), 4.42 (t, 1H, CH-NH), 1.79 (d, 2H, CH_2_); ^13^C NMR (101 MHz, DMSO-d_6_) δ (ppm): 165.12, 162.22, 159.13, 141.85, 135.13, 134.81, 132.65, 131.84, 129.44, 127.54, 123.43, 120.42, 117.20, 61.81, 34.20; IR (KBr) ν: 3457 (NH), 1682 (C=O), 1598 (C=N), 1270 (C–N); Anal. Calcd for C_35_H_26_N_4_O (518.21): C, 81.06%; H, 5.05%; N, 10.80%. Found: C, 80.86%; H, 4.88%; N, 10.64%.

##### 3-(4-(2-(4-chlorophenyl)-2,3-dihydro-1H-benzo[b][1,4]diazepin-4-yl)phenyl)-2-phenylquinazolin-4(3H)-one (3d)

Yield 77%; mp 175–177 °C; ^1^H NMR (400 MHz, DMSO-d_6_) δ (ppm): 11.62 (s, 1H, NH), 7.17–8.75 (m, 21H, Ar–H), 4.41 (t, 1H, CH-NH), 1.55 (d, 2H, CH_2_); ^13^C NMR (101 MHz, DMSO-d_6_) δ (ppm): 171.10, 168.30, 165.20, 141.60, 135.10, 134.80, 132.60, 131.80, 129.50, 127.59, 127.54, 123.40, 121.20, 120.40, 61.90, 30.70; IR (KBr) ν: 3630 (NH), 1681 (C=O), 1598 (C=N), 1231 (C–N); Anal. Calcd for C_35_H_25_ClN_4_O (553.05): C, 76.01%; H, 4.56%; Cl, 6.41; N, 10.13%. Found: C, 75.86%; H, 4.38%; Cl, 6.21; N, 10.03%.

##### 2-phenyl-3-(4-(2-styryl-2,3-dihydrobenzo[b][1,4]oxazepin-4-yl)phenyl)quinazolin-4(3H)-one (4a)

Yield 67%; mp 190–192 °C; ^1^H NMR (400 MHz, DMSO-d_6_) δ (ppm): 7.54–8.71 (m, 22H, Ar–H), 4.10 (m, 1H, CH-O), 1.89 (d, 2H, CH_2_); 7.15 (d, 1H, CH=CH-Ph), 7.36 (d,1H, CH=CH-Ph); ^13^C NMR (101 MHz, DMSO-d_6_) δ (ppm): 171.12, 168.57, 165.21, 141.95, 135.22, 134.33, 132.50, 131.51, 129.21, 127.70, 123.12, 120.22, 118.12, 75.23, 36.12; IR (KBr) ν: 1656 (C=O), 1589 (C=N), 1243 (C–O); Anal. Calcd for C_37_H_27_N_3_O_2_ (545.64): C, 81.45%; H, 4.99%; N, 7.70%. Found: C, 81.35%; H, 4.79%; N, 7.56%.

##### 3-(4-(2-(furan-3-yl)-2,3-dihydrobenzo[b][1,4]oxazepin-4-yl)phenyl)-2-phenylquinazolin-4(3H)-one (4b)

Yield 74%; mp 184–186 °C; ^1^H NMR (400 MHz, DMSO-d_6_) δ (ppm): 7.00–8.90 (m, 20H, Ar–H), 4.20 (t, 1H, CH-O), 1.91 (d, 2H, CH_2_); ^13^C NMR (101 MHz, DMSO-d_6_) δ (ppm): 170.90, 168.40, 165.10, 141.20, 135.20, 134.70, 132.50, 131.20, 129.80, 127.20, 124.20, 120.9, 116.20, 84.10, 36.20; IR (KBr) ν: 1688 (C=O), 1540 (C=N), 1233 (C–O); Anal. Calcd for C_33_H_23_N_3_O_3_ (509.55): C, 77.78%; H, 4.55%; N, 8.25%. Found: C, 77.46%; H, 4.35%; N, 8.15%.

##### 2-phenyl-3-(4-(2-phenyl-2,3-dihydrobenzo[b][1,4]oxazepin-4-yl)phenyl)quinazolin-4(3H)-one (4c)

Yield 80%; mp 162–164 °C; ^1^H NMR (400 MHz, DMSO-d_6_) δ (ppm): 7.05–8.81 (m, 22H, Ar–H), 4.50 (t, 1H, CH-O), 1.91 (d, 2H, CH_2_); ^13^C NMR (101 MHz, DMSO-d_6_) δ (ppm): 170.50, 165.20, 162.10, 140.80, 134.80, 134.50, 132.60, 131.80, 129.50, 127.70, 123.40, 120.40,117.20, 78.50, 32.40; IR (KBr) ν: 1688 (C=O), 1591 (C=N), 1230 (C–O); Anal. Calcd for C_35_H_25_N_3_O_2_ (519.59): C, 80.90%; H, 4.85%; N, 8.09%. Found: C, 80.70%; H, 4.58%; N, 7.94%.

##### 3-(4-(2-(4-chlorophenyl)-2,3-dihydrobenzo[b][1,4]oxazepin-4-yl)phenyl)-2-phenylquinazolin-4(3H)-one (4d)

Yield 65%; mp 166–168 °C; ^1^H NMR (400 MHz, DMSO-d_6_) δ (ppm): 7.05–8.89 (m, 21H, Ar–H), 4.20 (t, 1H, CH-O), 1.65 (d, 2H, CH_2_); ^13^C NMR (101 MHz, DMSO-d_6_) δ (ppm): 170.53, 168.14, 165.34, 141.95, 141.5, 134.82, 134.71, 132.7, 131.81, 131.26, 129.52, 127.61, 127.54, 123.89, 123.48, 121.39, 118.12, 117.54, 61.95, 31.12; IR (KBr) ν: 1679 (C=O), 1598 (C=N), 1234 (C–O); Anal. Calcd for C_35_H_24_ClN_3_O_2_ (554.05): C, 75.88%; H, 4.37%; Cl, 6.40%; N, 7.58%. Found: C, 75.66%; H, 4.18%; Cl, 6.24%; N, 7.44%.

### Molecular docking in-silico simulations

Molecular docking studies investigated the binding patterns of the ligand molecules to the target proteins outer membrane protein A (PDB ID: 2ge4) the exo-1,3-beta glucanase (PDB ID: 4m80), Smoothened (SMO) (PDB ID: 5L7D), and transcription factor glioma-associated homology (SUFU/GLI-1) (PDB ID: 4KMD).The 3D structure of the target proteins were retrieved from the protein bank database (PDB) and were prepared by removal of all water molecules, native crystallization legend and cofactors, then protonate using (MVD) software. The newly synthesized azepine derivatives based on quinazolinone moiety and reference drugs were drawn using Chemdraw ultra 8.0 (https://en.freedownloadmanager.org/users-choice/Chemdraw_Ultra_8.0.html), and energy was minimized using MM2 force field then saved in mol format. The computation molecular docking was performed using Molegro Virtual Docker (MVD) (http://www.molegro.com/mvd-product.php, 17–2-2021)^23^.

#### In-silico ADMET prediction

The online tool SwissADME (http://www.swissadme.ch/) from the Swiss Institute of Bioinformatics was utilized to investigate the pharmacokinetics and drug-likeness prediction of the newly synthesized compounds. The compound's 2D structural model was converted into SMILES using SwissADME's SMILES generator. The SMILES data was then examined to identify the compound's ADMET properties, including pharmacokinetics and drug-likeness^24,25^.

### Biological evaluations (in vitro)

#### Antimicrobial assessments

The clinical strains utilized in this experiment were graciously donated by the clinical laboratory at Tanta University Hospital in Tanta, Egypt. Four multidrug-resistant Gram −ve and Gram + ve bacterial strains *E. coli*, *P. aeuroginosa*, *S. aureus*, *B. subtilis* and two pathogenic unicellular fungi *C. Albicans* and *A. flavus* were used. The azepine derivatives which show the best inhibitory binding energies in the *in-silico* studies were proceeded for further antimicrobial investigations.

##### Antimicrobial activity testing of the novel azepine derivatives using agar well diffusion method

By using the agar well diffusion technique, the diameter of the inhibitory zone was determined to evaluate how susceptible the tested bacterial and fungal strains were to the synthetic novel azepine derivatives. The synthesized azepine derivatives were prepared in DMSO at a concentration of 10 mg/mL antibiotic Ciprofloxacin, antifungal Clotrimazole and DMSO were used as positive and negative controls, respectively, to compare the effectiveness of bacterial and fungal strains. Before being adjusted to 106 CFU/mL at 630 nm, the bacterial and fungal strains underwent an overnight sub-culture in a nutrient broth medium. A 100 μL aliquot of each broth culture was evenly seeded throughout the nutrient agar medium using a sterile disposable plastic rod. On the surface of the nutritional agar medium, 9 mm wells were successfully made using a sterile cork porer, and 50 μL of each compound was then added^26^. The % activity index for the complex was calculated by the formula as follow:

$$\mathrm{\%\,Activity\,Index}= \frac{\mathrm{Zone\,of\,inhibition\,by\,test\,compound }\,({\text{diametre}})}{\mathrm{Zone\,of\,inhibition\,by\,standard }\left({\text{diametre}}\right)}\times 100$$

##### Minimal inhibitory concentration (MIC)

The azepine derivatives were next evaluated in DMSO at different dosages (0.5, 3.75, 7.5, 10 mg/mL) to observe their antimicrobial properties. The examined bacterial or fungal strains were placed in a loop that was submerged in 10 mL of nutrient broth and grown at 30 °C overnight. Test tubes were prepared and sterilized with 9.5 mL of 10 × diluted nutritional broth. The tubes were inoculated with 0.5 mL of the suitable microbe that had been cultured overnight. The chosen bioactive azepine derivatives were added to the tubes containing the nutrient broth. A shaking incubator was used to stir the cultures of the test organisms and the synthesized azepines at various concentrations at 30 °C. Following 24 h, the number of living cells was determined as colony-forming units/milliliter (CFU/mL) in accordance with Nakashima et al.'s instructions^27^.

#### Antineoplastic and cytotoxic studies

##### Cell lines and culture conditions

Human normal lung fibroblast (WI-38; (#ATCC CCL-75), hepatocellular carcinoma (HepG-2;(# ATCC HB-8065), Colorectal carcinoma (HCT-116; (#ATCC CCL-247) and Michigan Cancer Foundation breast carcinoma (MCF-7; (#ATCC HTB-22). The cell lines were purchased from ATCC via VACSERA, Cairo, Egypt. These cell lines were seeded at a density of 1 × 10^4^ cells/well using DMEM media with 10% FBS and 1% penicillin/streptomycin and incubated at 37 °C for 24 h under 5% CO_2_^28^.

##### Cells treatment and viability assay

Different concentrations (0–200 μM) of both the newly azepine derivatives and the reference inhibitory Hedgehog GANT-61 drug were applied to the cells. MTT (5 mg/mL in PBS) was applied to each well after 48 h of incubation, and the cells were then cultivated for an additional 4 h at 37 °C in a cell culture incubator. 100 μL of DMSO was added to the wells after supernatant aspiration and shaken for 15 min. Using a microplate reader (Bio-Rad, CA, USA), the absorbance was found at 630 nm^17^.

### Statistical analysis

The experimental data were expressed as the mean ± SE, and the IC_50_ values were calculated Nonlinear regression curve fit (dose–response inhibition) using GraphPad Prism software 6 (San Diego, CA) (https:// www.graph pad. com/ scientific- software/ prism/).

## Results and discussion

### Chemistry of the synthesized compounds

**Figure 2:**
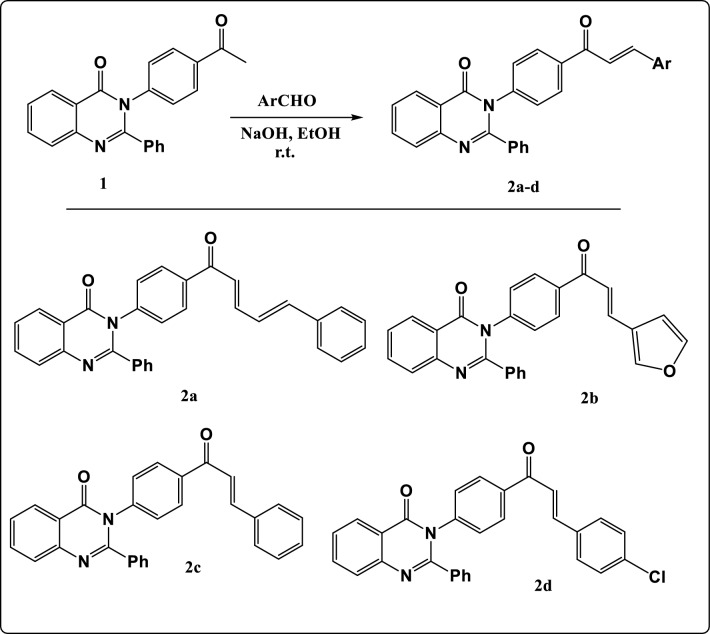
Synthesis pathway of compounds (**2a–d**).

A series of chalcones (**2a–d**) were synthesized through the one pot reaction between 3-(4-acetylphenyl)-2-phenylquinazolin-4(3H)-one (**1**) with different aromatic aldehydes as illustrated in Fig. 2. The structure of these compounds was confirmed via elemental analysis and different spectroscopic data.

**Figure 3:**
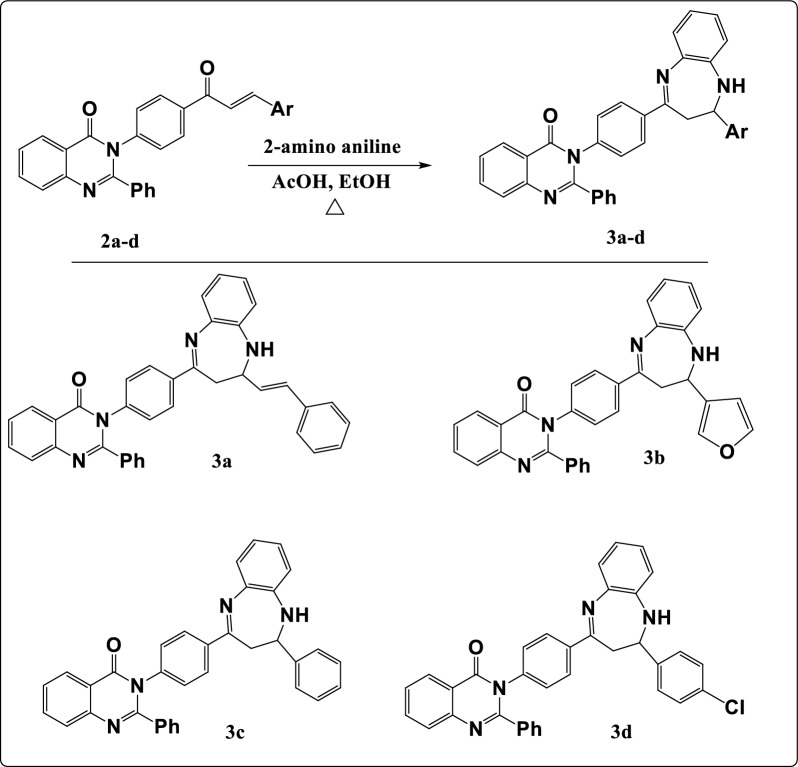
Synthesis pathway of diazepines (**3a–d**).

According to Fig. 3, the reaction of chalcones **(2a–d)** with 2-amino aniline offers the diazepine derivatives **(3a–d)**. Their chemical structures were demonstrated via both elemental analysis and different spectral data. The FT-IR spectra showed an absorption band at 1537–1611 cm^−1^ which characterized to C=N of diazepine ring and at 3300–3630 cm^−1^ for NH group which appeared as a singlet signal at δ 10.65–11.72 ppm in ^1^H-NMR spectra. Revealed a new signal resonated at δ 3.35–4.42 ppm due to CH proton of diazepine ring and a doublet signal at δ 1.55–2.18 ppm attributed to CH_2_ group. ^13^C-NMR spectra displayed signals at δ 164.40–171.10, δ 58.20–61.90 and δ 30.70–34.40 due to C=N, CH and CH_2_ respectively in the diazepine ring.

**Figure 4:**
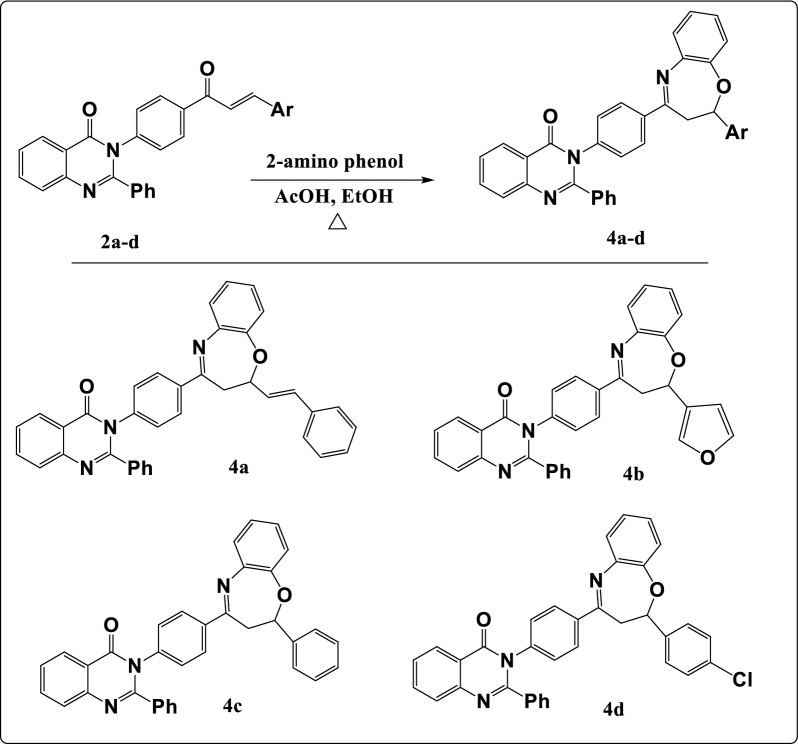
Synthesis pathway of oxazepines (**4a–d**).

Further modification of compound **1** with *o*- aminophenol offered oxazepine derivatives **(4a–d)** as illustrated in Fig. 4. Their FT-IR spectra indicated the appearance of absorption band at 1540–1598 cm^−1^ for C=N of oxazepine ring. The ^1^H-NMR spectra offered a signal at δ 4.10–4.50 ppm resonated to CH proton of oxazepine ring and doublet signal at 1.65–1.91 ppm for CH_2_ group. ^13^C-NMR spectra offered signal at δ 170.50–171.12, δ 61.95–84.10 and δ 31.12–36.20 for C=N, CH and CH_2_ respectively in the oxazepine ring.

### In silico docking investigations

**Figure 5:**
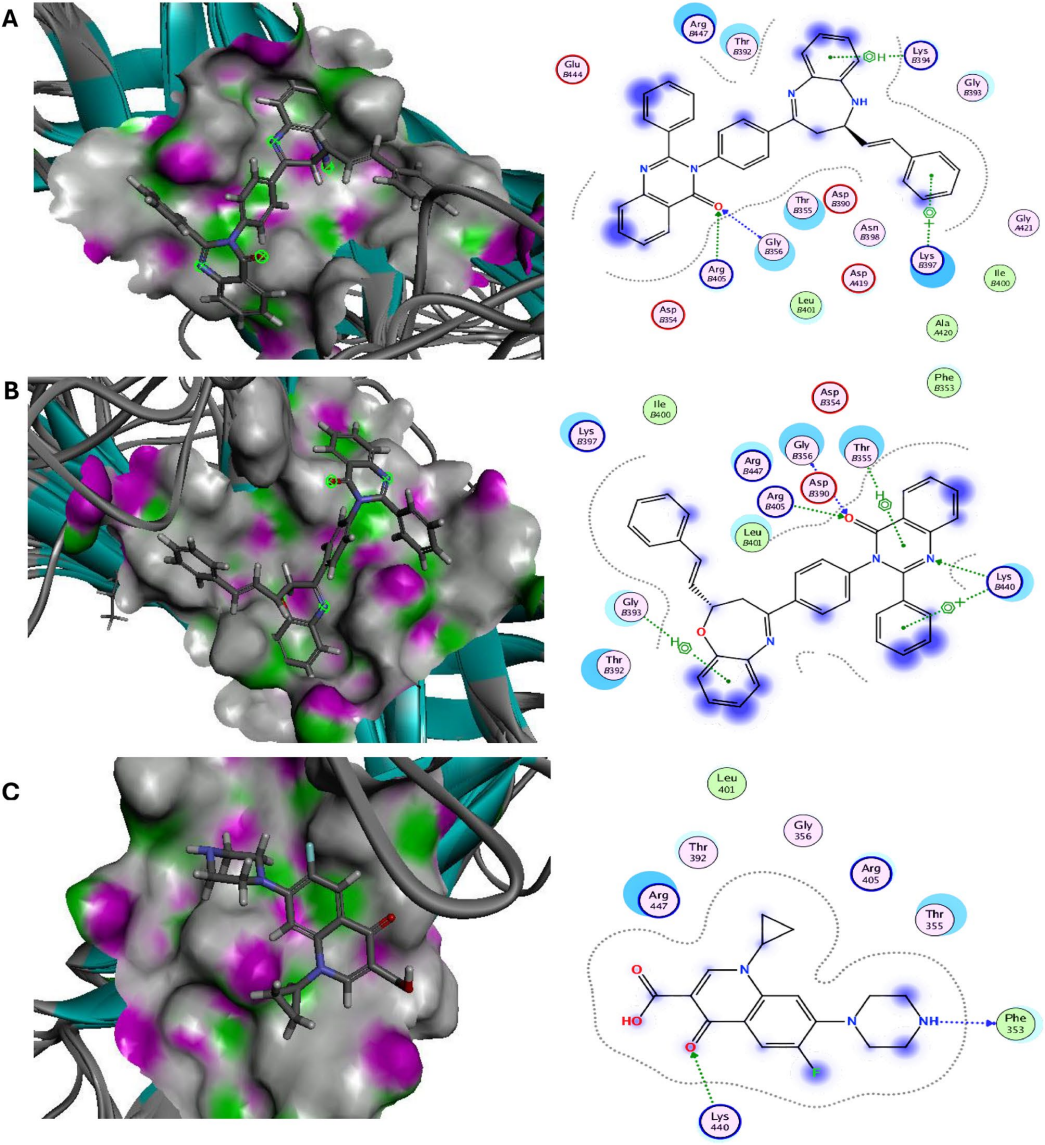
Molecular docking interactions of the best binding energy (**A**) Diazepine **(3a), **(**B**) Oxazepine **(4a)**, and (**C**) reference drug with OmpA protein. 3D-(Left side) and 2D-(Right side).

**Figure 6:**
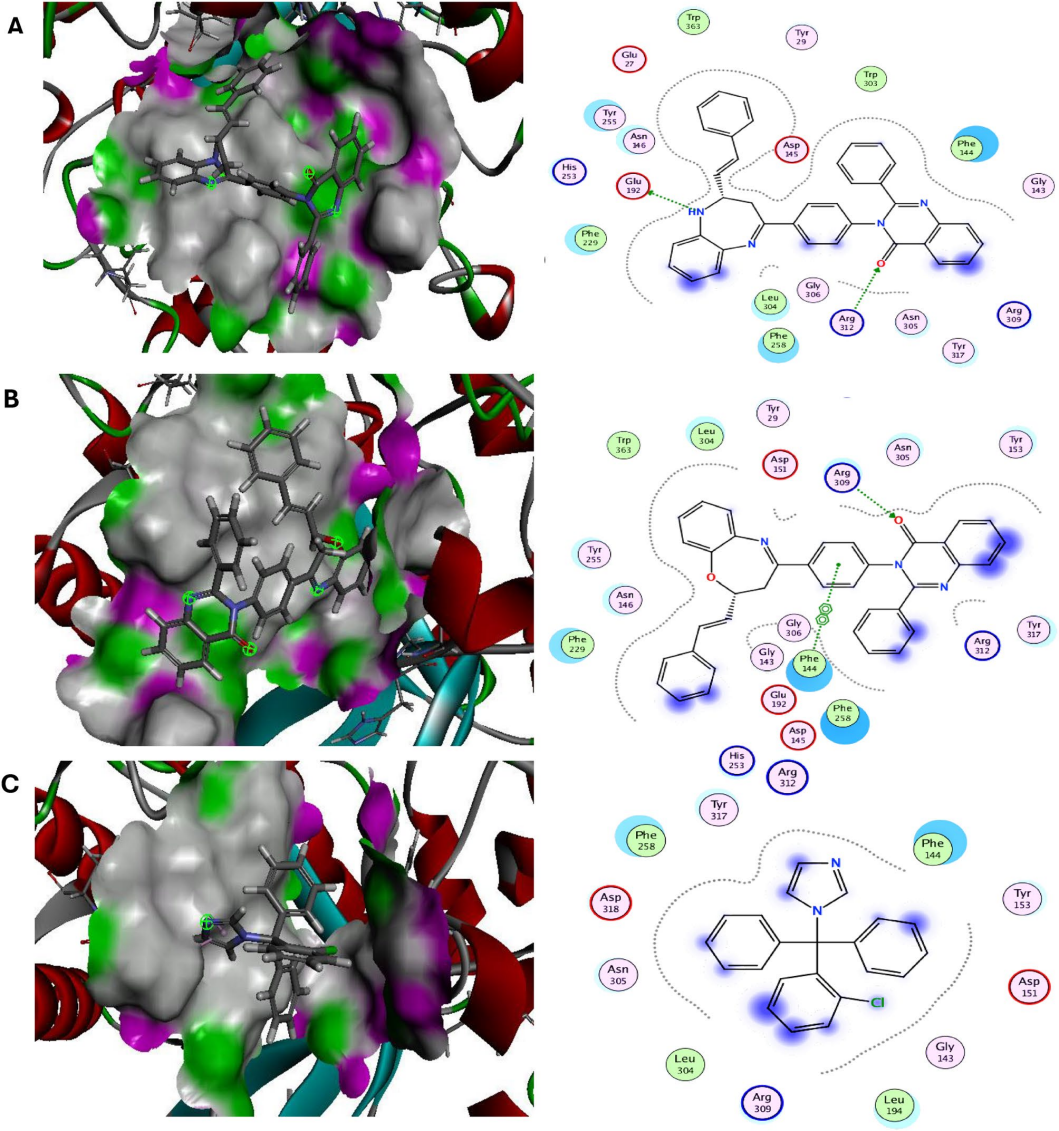
Molecular docking interactions of the best binding energy (**A**) Diazepine **(3a)**, (**B**) Oxazepine **(4a)**, and (**C**) reference drug with exo-1,3-beta-glucanase protein. 3D-(Left side) and 2D -(Right side).

Molecular docking has been widely manipulated for the discovery of novel medications as it is an effective method for rapidly and accurately predicting protein–ligand complex binding energies and biomolecular conformations^29^. Herein, the novel diazepines ligands **(3a–3d)** and oxazepines ligands **(4a–4d)** were docked into outer membrane protein A (OMPA) and exo-1,3-beta-glucanase are well-known, appealing therapeutic target proteins for the development of antibacterial and antifungal drugs^30,31^. The molecular dock score (Mol dock score) was used to express the binding affinity of the docked molecules as negative binding energy kcal/mol. The ligands with a more negative Mol Dock score will have a higher affinity for protein binding. All novel azepines interactions with target antimicrobial proteins were described in Table S1 and the top ranked compounds were elucidated in Figs. 5, 6 and Table 1. Our results elucidated that diazepine **(3a)** and oxazepine **(4a)** showed the highest binding energy against target OMPA and exo-1,3-beta-glucanase with values equal to − 7.54, − 7.73 and − 8.23, − 7.87 kcal/mol, compared with the reference antibiotic Ciprofloxacin and antifungal Clotrimazole (− 6.95, − 6.23 kcal/mol), respectively. Diazepine **(3a)** binds via hydrogen interactions with the OMPA essential residues GLYB356, ARGB405, π interactions with LYSB394, LYSB397 and electrostatic interactions with ARGB447, THRB392, GLYB393, ASNB398, THRB355, LEUB401, GLYB444, ASPA419. Moreover, oxazepine **(4a)** binds via hydrogen interactions with the OMPA essential residues ARGB405, GLYB356, LYSB440 π interactions with THRB355, LYSB440, GLYB393 and electrostatic interactions with LYSB397, LEUB401, ARGB447, ASPB390, PHEB353, THRB392 compared with the antibiotic Ciprofloxacin reference drug that binds via hydrogen interactions with the OMPA essential residues LYS440, PHE353 and electrostatic interactions with ARG447, THR392, ARG405, THR355. Further, Diazepine **(3a)** binds via hydrogen interactions with the exo-1,3-beta-glucanase essential residues GLU192, ARG312 and electrostatic interactions with PHE229, ASN146, TYR255, TRP363, PHE144, TYR317, ASN305, LEU304, PHE258. Furthermore, oxazepine **(4a)** binds via hydrogen interactions with the exo-1,3-beta-glucanase essential residue ARG309, π interaction with PHE144 and electrostatic interactions with LEU304, TYR29, ASN305, TYR153, TYR317, PHE258, ASN146, PHE229, TYR255 compared with the antifungal Clotrimazole reference drug that binds via electrostatic interactions with PHE258, TYR317, PHE144, TYR153, LEU194, ARG309, ASN305, ASP318.

**Table 1:** Calculated docking scores (kcal/mol) of compounds **3a** and **4a** and reference drugs with the target proteins.

Comps	Antimicrobial target proteins				Hedgehog signaling target proteins			
	OMPA		Exo-1,3-beta-glucanase		SMO		SUFU/GLI-1	
	Docking Score (ΔGbind)	Docked complex (amino acid– ligand) interactions	Docking Score (ΔGbind)	Docked complex (amino acid– ligand) interactions	Docking Score (ΔGbind)	Docked complex (amino acid–ligand) interactions	Docking Score (ΔGbind)	Docked complex (amino acid–ligand) interactions
**3a**	− 7.54	*Hydrogen interaction* GLYB356 ARGB447 *π interaction* LYSB397 LYSB394 *Electrostatic interaction* THRB392 GLYB393 ASNB398 THRB355 LEUB401 GLYB444 ASPA419	− 8.23	*Hydrogen interaction* ARG312 GLU192 *Electrostatic interaction* PHE229 ASN146 TYR255 TRP363 PHE144 TYR317 ASN305 LEU304 PHE258	− 8.02	*π interaction* ARGB451 PHEA523 *Electrostatic interaction* ILEB454 TYRA233 THRB534 GLUB447 TRPB537 THRB448 THRB538 PHEA237 VALA240 ALAA236	− 8.90	*Hydrogen interaction* THRA205 LYSA457 *Electrostatic interaction* GLNA175 HISA209 GLNA375 HISA164 GLUA376 ASPA476 LEUA458 GLUA455 PROA453 PHEA456 GLUA454
**4a**	− 7.73	*Hydrogen interaction* ARGB405 GLYB356 LYSB440 *π interaction* LYSB440 THRB355 GLYB393 *Electrostatic interaction* LYSB397 LEUB401 ARGB447 ASPB390 PHEB353 THRB392	− 7.87	*Hydrogen interaction* ARG309 *π interaction* PHE144 *Electrostatic interaction* LEU304 TYR29 ASN305 TYR153 TYR317 PHE258 ASN146 PHE229 TYR255	− 7.96	*Hydrogen interaction* ARGB451 *π interaction* PHEA523 LYSB539 *Electrostatic interaction* THRB448 LEUB542 TRPB537 GLUB447 PHEB455 ILEB454 TYRA233 PHEA237 THRB538	− 7.95	*π interaction* THRB128 HISA164 *Electrostatic interaction* GLUA455 PROA453 GLNA375 HISA209 SERA165 GLUA454 TRPA163 GLNA212 GLUA376 THRA205 GLYB127
**Ciprofloxacin (Reference antibiotic)**	− 6.95	*Hydrogen interaction* PHE353 LYS440 *Electrostatic interaction* ARG447 THR392 ARG405 THR355	–	–	–	–	–	–
**Clotrimazole (Reference antifungal)**	–	–	− 6.23	*Electrostatic interaction* PHE258 TYR317 PHE144 TYR153 LEU194 ARG309 ASN305 ASP318	–	–	–	–
**GANT-61 (Reference HH-GLI)**	–	–	–	–	− 7.62	*Hydrogen interaction* ARGB451 *Electrostatic interaction* THRB538 TYRA233 ILEB454 PHEA523 THRB534 PHEA237 TRPB537 GLUB447 LEUB458	− 6.95	*π interaction* HISA164 GLUA455 GLUA454 *Electrostatic interaction* PROA453 THRA205 GLNA375 THRB128

**Figure 7:**
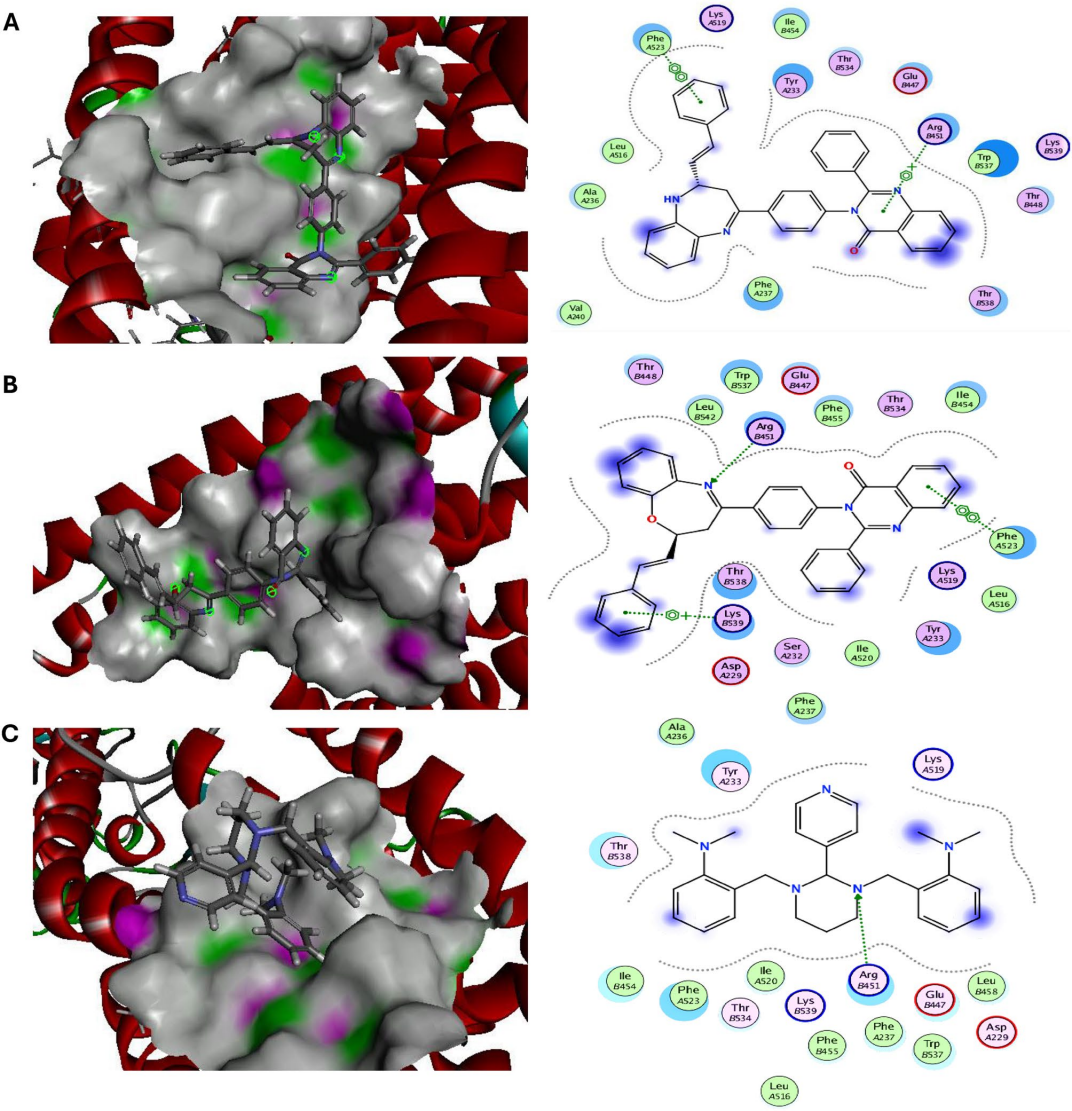
Molecular docking interactions of the best binding energy (**A**) Diazepine **(3a)**, (**B**) Oxazepine **(4a)**, and (**C**) reference drug with SMO protein. 3D-(Left side) and 2D -(Right side).

**Figure 8:**
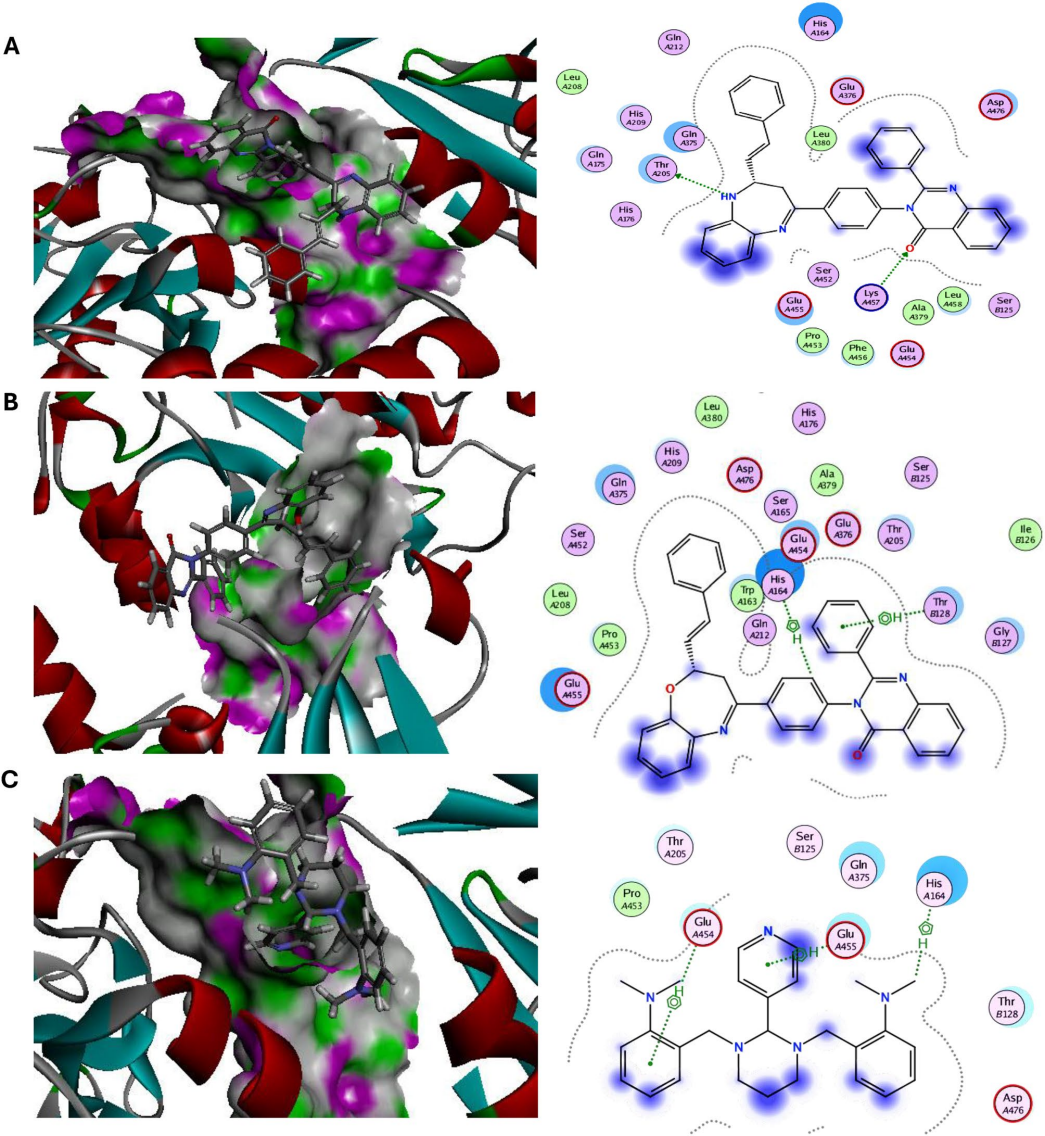
Molecular docking interactions of the best binding energy (**A**) Diazepine **(3a)**, (**B**) Oxazepine **(4a)**, and (**C**) reference drug with SUFU/GLI-1 protein. 3D-(Left side) and 2D -(Right side).

Additionally, it has been demonstrated that the hedgehog (HH-GLI) signaling pathway controls cellular differentiation, migration, and maintenance of tissue progenitor cells during wound healing and regeneration processes. Therefore, aberrant activation of this signal in cancer cells leads to malignant transformations such as dedifferentiation, acquisition of stemness features, and migration potency^32^. Exclusively, the novel diazepines ligands **(3a–3d)** and oxazepines ligands **(4a–4d)** were docked with Smoothened (SMO), transcription factor glioma-associated homology (SUFU/GLI-1), the main proteins of Hedgehog signaling pathway to inspect their anticancer potential. All novel azepines interactions with target HH-GLI proteins were described in were described in Table S1 and the top ranked compounds were elucidated in Figs. 7, 8 and Table 1. Our results observed that, diazepine **(3a)** and oxazepine **(4a)** showed the highest binding energy against target SMO and SUFU/GLI-1 with values equal to − 8.02, − 7.96 and − 8.90, − 7.95 kcal/mol compared with the reference HH-GLI inhibitor GANT-61 (− 7.62 and − 6.95 kcal/mol) respectively. Diazepine **(3a)** binds via π-π interactions with the SMO essential residues PHEA523, ARGB451, and binds via electrostatic interactions with ILEB454, TYRA233, THRB534, GLUB447, TRPB537, THRB448, THRB538, PHEA237, VALA240, ALAA236. Besides, oxazepine **(4a)** binds via hydrogen interactions with the SMO essential residue ARGB451, π interactions with PHEA523, LYSB539 and binds via electrostatic interactions with THRB448, LEUB542, TRPB537, GLUB447, PHEB455, ILEB454, TYRA233, PHEA237, THRB538 compared with reference GANT-61 HH inhibitor that binds with SMO target protein via hydrogen interaction with ARGB451 and electrostatic interactions with TYRA233, THRB538, ILEB454, PHEA523, THRB534, PHEA237, TRPB537, GLUB447, LEUB458. Also, diazepine **(3a)** binds via hydrogen interactions with the SUFU/GLI-1 essential residues THRA205, LYSA457 and binds via electrostatic interactions with GLNA175, HISA209, GLNA375, HISA164, GLUA376, ASPA476, LEUA458, GLUA455, PROA453, PHEA456, GLUA454. While oxazepine **(4a)** binds via π-π interactions with SUFU/GLI-1 essential residues HISA164, THRB128 and binds via electrostatic interactions with GLUA455, PROA453, GLNA375, HISA209, SERA165, GLUA454, TRPA163, GLNA212, GLUA376, THRA205, GLYB127 compared with GANT-61 HH inhibitor that binds with SUFU/GLI-1 target protein via π-π interactions with HISA164, GLUA455, GLUA454 and binds via electrostatic interactions with PROA453, THRA205, GLNA375 THRB128. Thus, our findings strongly state that diazepine **(3a)** and oxazepine **(4a)** have antimicrobial and anticancer impact.

#### Studies on ADMET pharmacokinetics features

**Figure 9:**
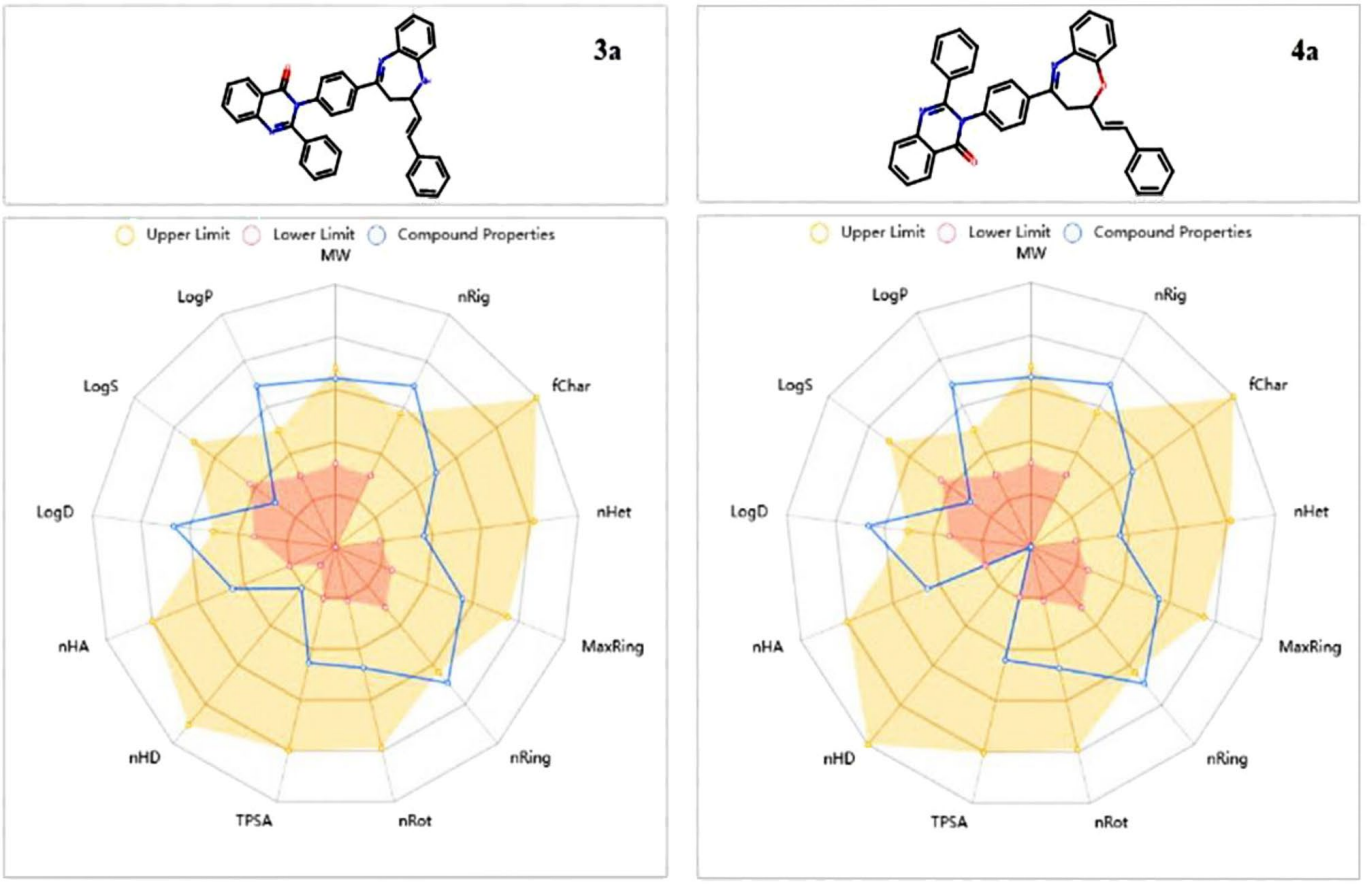
ADMET pharmacokinetics features of Diazepine **(3a)** and Oxazepine **(4a)**.

ADMET must certify the drug's effectiveness as a top candidate against any disease. Using in-silico Physio-chemical methods, the partition coefficient (cLogP), donor hydrogen bond, and drug similarity were all computed. Additionally, pharmacokinetic and bioavailability investigations have been conducted to carry out such clinical studies on these newly synthesized oxazepine (**4a**) and diazepine (**3a**). To have excellent oral bioavailability, the topological polar surface (TPSA) should be less than ˂ 140 Å2. Based on our findings, the TPSA for diazepine (**3a**) and oxazepine (**4a**) were 59.28 and 56.48, respectively. Furthermore, the findings demonstrated that oxazepine (**4a**) and diazepine (**3a**) had no BBB which demonstrated their CNS protection also they had good gastrointestinal absorptions. For the newly synthesized candidate to be considered for development, it must first pass a toxicity risk assessment. AMES toxicity analysis was conducted, and diazepine **(3a)** and oxazepine **(4a)** tested negative, indicating that they have no mutagenic toxic effects. Surprisingly, none of the substances proved carcinogenic, this prompted an *in-silico* investigation, the results of which are shown in Table 2, Fig. 9. Based on our findings, the best-docked diazepine **(3a)** and oxazepine **(4a)**, which also greatly inhibit target proteins, demonstrated suitable physio-chemical, pharmacokinetic, and bioavailability in silico without any toxicity or carcinogenicity, suggesting that they might be a promising new class of antimicrobial and anticancer drugs.

**Table 2:** Pharmacokinetic properties.

	Molecular Weight (g/mol)	Blood–Brain Barrier (Log BBB)	%Human Intestinal Absorption (HIA +)	TPSA A2	Log p	HBA	HBD	N rotatable	GI absorption	AMES toxicity	Carcinogenicity
Acceptable ranges	≤ 500	> 0.3 great < − 1 poor	> 80% high < 30% low	≤ 140	< 5	2.0–20.0	0.0–6.0	≤ 10		Nontoxic	Noncarcinogenic
**3a**	544.23	− 0.179	99.73	59.28	4.88	5	1	5	High	Nontoxic	Noncarcinogenic
**4a**	545.63	− 0.217	98.32	56.48	4.92	5	0	5	High	Nontoxic	Noncarcinogenic

### In vitro biological assessments

#### Antimicrobial studies

**Figure 10:**
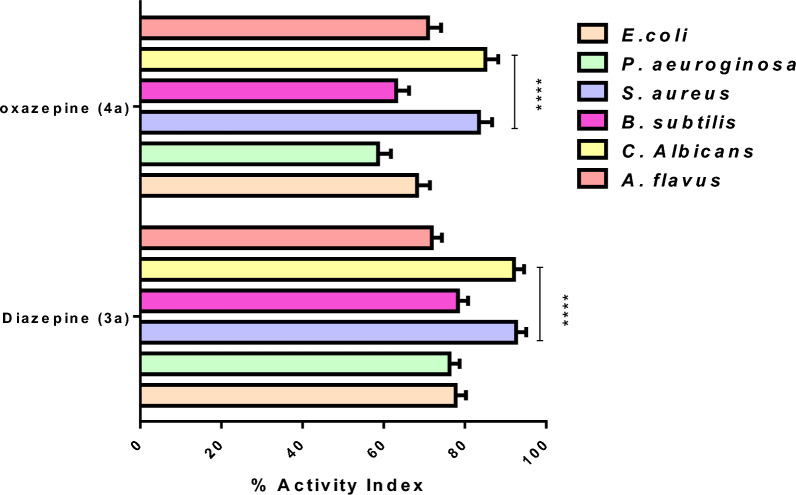
Percentage of antimicrobial activity index for diazepine **(3a)** and oxazepine **(4a)** against six pathogenic strains, ****p < 0.0001 significantly vs all microbial strains.

Resistant strains have evolved as a significant threat to population health and the global economy because of reckless antibiotic usage and inadequate infection management. As a result, it is critical to conduct extensive research and develop a new class of antimicrobial compounds to halt the spread of antimicrobial resistance (AMR)^33^. According to the *in-silico* results, diazepine **(3a)** and oxazepine **(4a)** were evaluated for *in-vitro* antibacterial activity against Gram-positive bacteria *S. aureus* (MTCC-96) and *B. subtilis* (MTCC-441), Gram-negative bacteria *E. coli* (MTCC-614) and *P. aeruginosa* (MTCC-1035) and fungal *C. albicans* (MTCC-3017) and *A. flavus* (MTCC-227) using a standard agar well diffusion method and inhibitory zone diameters (mm) are summarized in Table 3; Fig. 10. Our results evaluated that diazepine **(3a)** and oxazepine **(4a)** elucidated strongest antimicrobial effect, inhibiting the growth of all the investigated microorganisms. These compounds generated significantly (*p* < 0.0001) the largest inhibition zones with *S. aureus* and *C. Albicans* (21.3, 22.1 and 19.2, 20.4 mm, respectively).

**Table 3:** Inhibitory zone diameters of compounds **3a** and **4a**.

Compounds	*E. coli*	*P. aeruginosa*	*S. aureus*	*B. subtilis*	*C. Albicans*	*A. flavus*
Diameter of inhibition zone (mm)						
** Diazepine (3a)**	17.1 ± 2.3	16 ± 2.1	21.3 ± 2.4	18 ± 1.1	22.1 ± 2.4	16 ± 1.0
** Oxazepine (4a)**	15 ± 2.0	12.3 ± 1.8	19.2 ± 1.3	14.5 ± 1.6	20.4 ± 3.1	15.6 ± 1.5
** Antibiotic ciprofloxacin reference drug**	22 ± 1.6	21 ± 2.2	23 ± 1.9	23 ± 2.7	–	–
** Antifungal clotrimazole reference drug**	–	–	–	–	24 ± 0.9	22 ± 2.0

Moreover, the smallest amount of the target diazepine **(3a)** and oxazepine **(4a)** required to inhibit microbial growth is referred to as the minimum inhibitory concentration (MIC). Drug formulations can benefit greatly from this approach. The ratio of surviving cell numbers was then assessed to estimate the level of antimicrobial activities of the diazepine **(3a)** and oxazepine **(4a)** (Table 4). diazepine **(3a)** and oxazepine **(4a)** exhibited reasonable biocidal activity at very low concentrations against the tested microorganisms compared with the reference drugs.

**Table 4:** MIC concentrations of newly azepine derivatives.

Compounds	*E. coli*	*P. aeruginosa*	*S. aureus*	*B. subtilis*	*C. Albicans*	*A. flavus*
MIC concentrations (mg/mL)						
** Diazepine (3a)**	2 ± 0.7	1.8 ± 1.0	1.3 ± 0.41	3.2 ± 0.65	1.6 ± 0.17	4.1 ± 0.25
** Oxazepine (4a)**	3.1 ± 0.52	2.3 ± 0.23	2 ± 0.13	4.5 ± 0.38	2 ± 0.26	5.2 ± 0.41
** Antibiotic ciprofloxacin reference drug**	0.5 ± 0.12	1 ± 0.05	0.5 ± 0.062	2 ± 0.14	–	–
** Antifungal clotrimazole reference drug**	–	–	–	–	1 ± 0.13	2 ± 0.21

#### In-vitro Antineoplastic cytotoxic studies

The antitumoral investigations using MTT assay were carried out to confirm the capability of diazepine **(3a)** and oxazepine **(4a)** in suppressing the aberrant of hedgehog (HH-GLI) signaling pathway, these azepine derivatives were selected among the others due to their highest binding energies in the *in-silico* studies. The MTT assay is a typical colorimetric method for evaluating cell growth. It is used to assess the cytotoxicity of other hazardous compounds and potential therapeutic medications. shortly, the enzyme mitochondrial dehydrogenases convert the yellow MTT into the purple formazan in living cells. By incorporating a suitable solvent, this formazan product is dissolved into a vibrant solution^34^. The precise concentration of the colored solution can be determined by measuring it at a particular wavelength. By plotting a dosage response curve and comparing the quantity of purple formazan that treated cells and untreated control cells generate, it is possible to evaluate how effectively the newly azepine derivatives destroy cancer cells.

**Figure 11:**
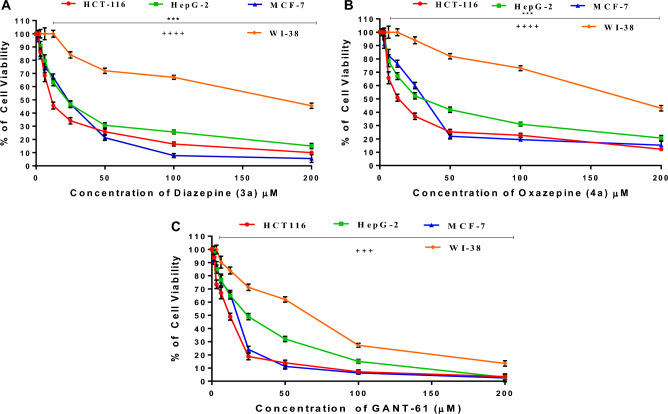
Antineoplastic cytotoxic dose–response curves. (**A**) Diazepine **(3a)**, (**B**) Oxazepine **(4a)**, and (**C**) GANT-61 (HH) reference inhibitor drug. ^***^p < 0.001 significantly vs standard GANT-61 effect on cancer cell lines, ^++++^p < 0.0001 and ^+++^p < 0.001 significantly vs the normal cell line.

Our results elucidated that the novel diazepine **(3a)** and oxazepine **(4a)** observed significant (*p* < 0.001) antitumor effect against panel of cancer cells (HCT-116, HepG-2 and MCF-7) compared with the reference HH-GLI inhibitory drug GANT-61. Moreover, the most crucial step in evaluating the anti-cancer effects of newly azepine derivatives is to determine their cytotoxicity on normal cell lines. Here, we used the human normal lung fibroblast (WI-38). Our findings showed that none of our newly synthesized azepine derivatives have any cytotoxicity on normal cells (*p* < 0.0001), in contrast to GANT-61, which showed moderate toxicity towards normal cells (*p* < 0.001) (Fig. 11; Table 5). The newly synthesized diazepine (3a) and oxazepine **(4a)** could therefore be employed as promising anticancer drugs by inhibiting HH-GLI signaling pathway. Therefore, the MTT results supported the outcomes of the molecular docking simulations.

**Table 5:** Cytotoxic activity of novel azepine derivatives against cancer and normal cell lines.

Compounds	*In-vitro* Cytotoxicity IC50 (μM)*			
	WI38	HCT-116	HepG-2	MCF-7
**Anticancer GANT-61 (HH) reference inhibitor**	55.03 ± 3.1	10.19 ± 0.3	21.90 ± 0.2	14.67 ± 0.2
**Diazepine (3a)**	168.08 ± 3.5	14.25 ± 1.1	24.57 ± 1.8	19.21 ± 0.8
**Oxazepine (4a)**	172.04 ± 3.7	16.95 ± 2.4	34.23 ± 2.0	28.28 ± 1.4

*IC_50_ (μM): 1–10 (very strong). 11–30 (strong). 31–60 (moderate). 61–100 (weak) and above 100 (non-cytotoxic).

### SAR (structure antimicrobial and anti-cancer activity relationship)

**Figure 12:**
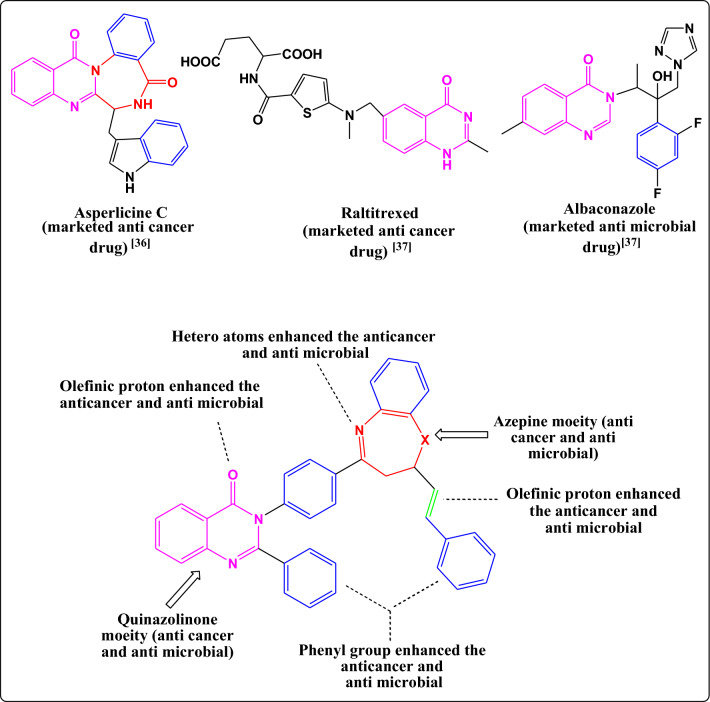
Structure activity relation of the novel synthesized azepine derivatives.

As described in Fig. 12 quinazolinones and azepines are found in many marketed anti-microbial and anti-cancer drugs^35,36^, both *in-silico* and *in-vitro* studies demonstrated the following SAR of the newly synthesized azepines:

The presence of different substituents in the aryl group enhanced both cytotoxic activity against different cancer cell lines (HCT-116, HepG-2, MCF-7) and antimicrobial activity against different types of Gram-positive bacteria (*S. aureus* (MTCC-96) and *B. subtilis* (MTCC-441)), Gram-negative bacteria (*E. coli* (MTCC-614) and *P. aeruginosa* (MTCC-1035)) and fungal (*C. albicans* (MTCC-3017) and *A. flavus* (MTCC-227)).

The presence of hetero atoms like oxygen and nitrogen in the chemical structure of the azepines enhanced the value of binding energy through the interaction between the azepines and the target protein via hydrogen bonding**.**

Compounds **3a** and **4a** were considered the most active compounds due to the presence of phenyl group and olefinic protons which enhanced the hydrogen bonding with the target protein and hence increased the value of the binding energy^18^.

## Conclusion

In conclusion, quinazolinone chalcones **(2a–d)** were synthesized and used for the synthesis of novel diazepines **(3a–d)** and oxazepines **(4a–d)**. The synthesized compounds were characterized by elemental analysis and different spectroscopic data. The results of novel diazepine **(3a)** and oxazepine **(4a)** antimicrobial effectiveness against six Gram-negative, positive multidrug-resistant bacterial isolates and unicellular pathogenic fungal strains showed a broad spectrum of their biocidal activity, which was consistent with their in-silico binding energies against OMPA and exo-1,3-beta-glucanase target proteins. Moreover, a substantial anticancer effect of diazepine **(3a)** and oxazepine **(4a)** were also observed against panel of cancer cell lines via suppressing hedgehog (HH-GLI) signaling pathway, which was supported by their molecular docking investigations against the SMO and SUFU/GLI-1 target (HH/GLI) proteins. Overall, it is recommended to use these novel diazepine **(3a)** and oxazepine **(4a)** as potential antimicrobial and anticancer agents in medical applications.

### Supplementary Information

Supplementary Information.

## Data Availability

The datasets generated and/or analyzed during the current study are available in: Macromolecule protein structure can be deposited in the worldwide protein data bank repository, (PDB IDs: 2ge4, 4m80, 5L7D, 4KMD). All cell lines were purchased from the American Type Culture Collection (ATCC) organization (# ATCC HB-8065, #ATCC CCL-247, #ATCC HTB-22, #ATCC CCL-75).
