# Supplementary material for: Synthesis and molecular docking simulations of novel azepines based on quinazolinone moiety as prospective antimicrobial and antitumor hedgehog signaling inhibitors
Source: Sci Rep. 2024 Feb 12;14:3530. doi: 10.1038/s41598-024-53517-y (PMC10861550; doi:10.1038/s41598-024-53517-y)
Supplement: Supplementary file 1 — Supplementary Information. [file 41598_2024_53517_MOESM1_ESM.pdf]

Supplementary Material

Synthesis and Molecular Docking Simulations of Novel Azepines based on Quinazolinone Moiety as Prospective Antimicrobial and Antitumor Hedgehog Signaling Inhibitors

Ahmed A. Noser[1*], A. A. El-Barbary[1], Maha M. Salem[2], Hayam A. Abd El Salam [3], Mohamed shahien[1]

[1]Organic Chemistry, Chemistry Department, Faculty of Science, Tanta University, Tanta 31527, Egypt.

[2]Biochemistry Division, Chemistry Department, Faculty of Science, Tanta University, Tanta 31527, Egypt.

[3]Green Chemistry Department, National Research Centre, Dokki, Giza, 12622, Cairo, Egypt.

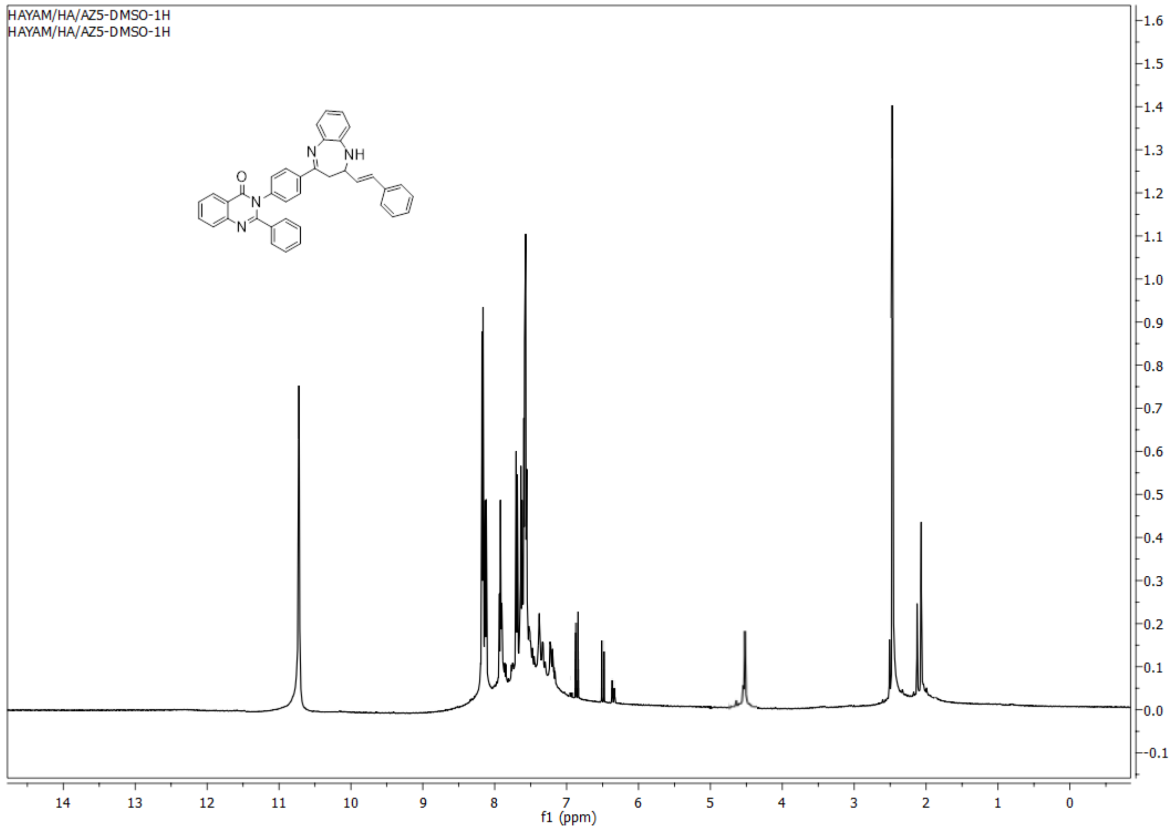

Figure S1: ^{1}H-NMR of compound 3a

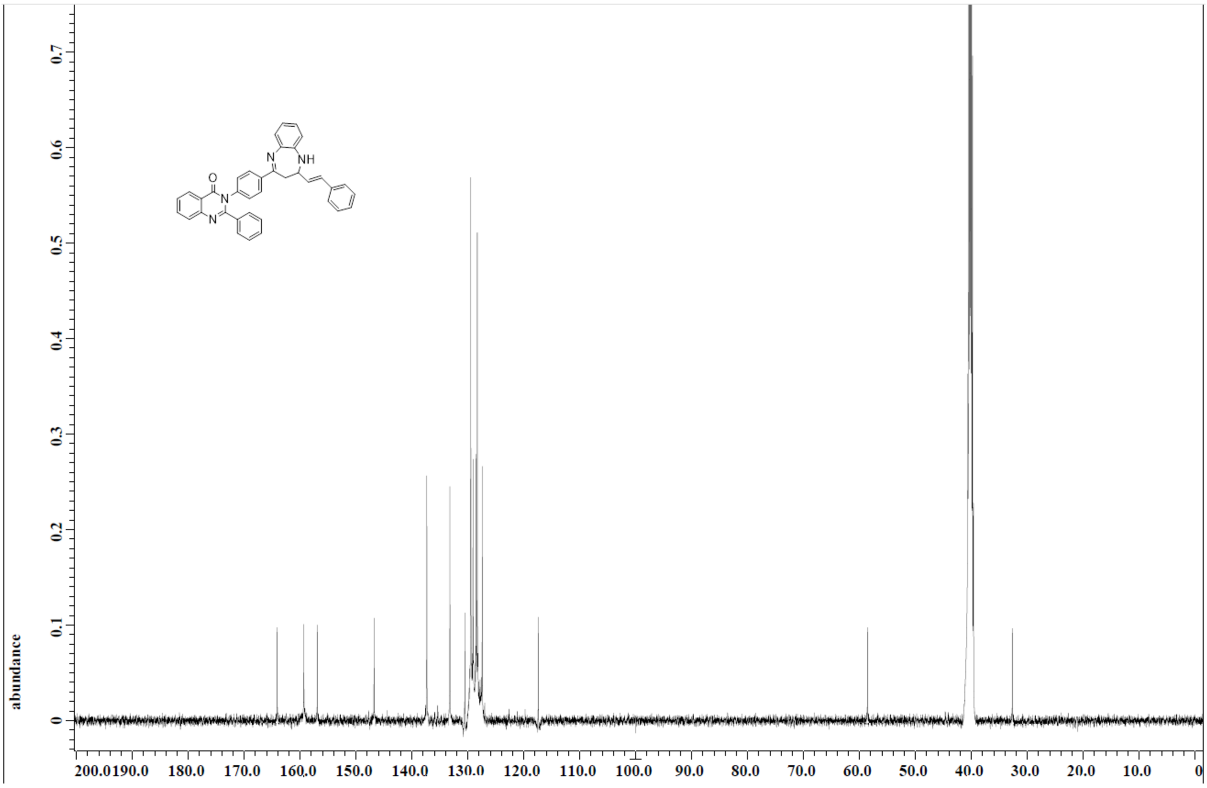

Figure S2: ^{13}C-NMR of compound 3a

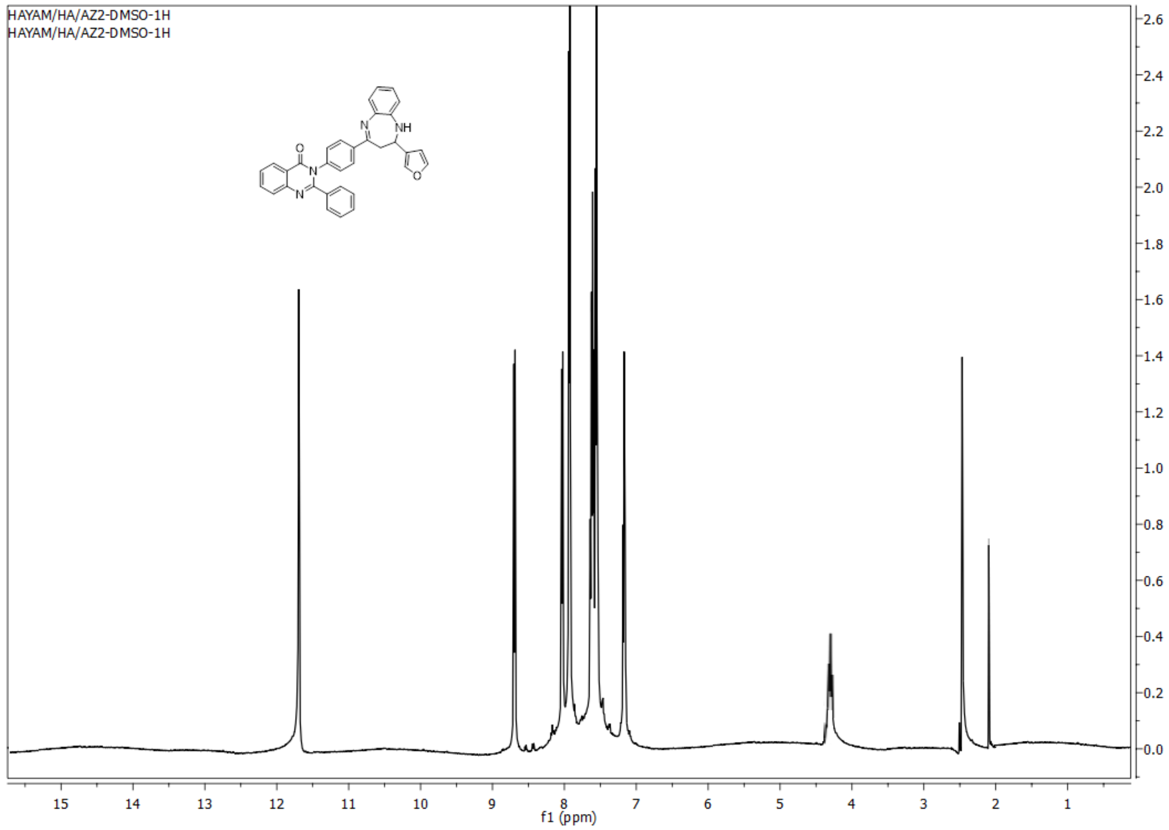

Figure S3: ^{1}H-NMR of compound 3b

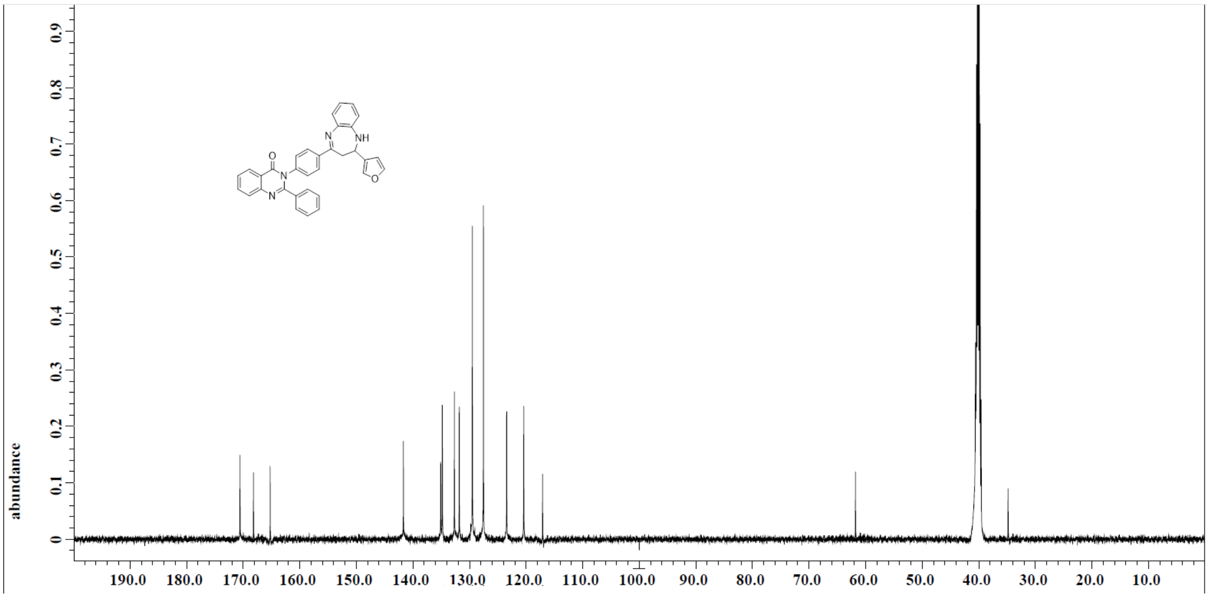

Figure S4: ^{13}C-NMR of compound 3b

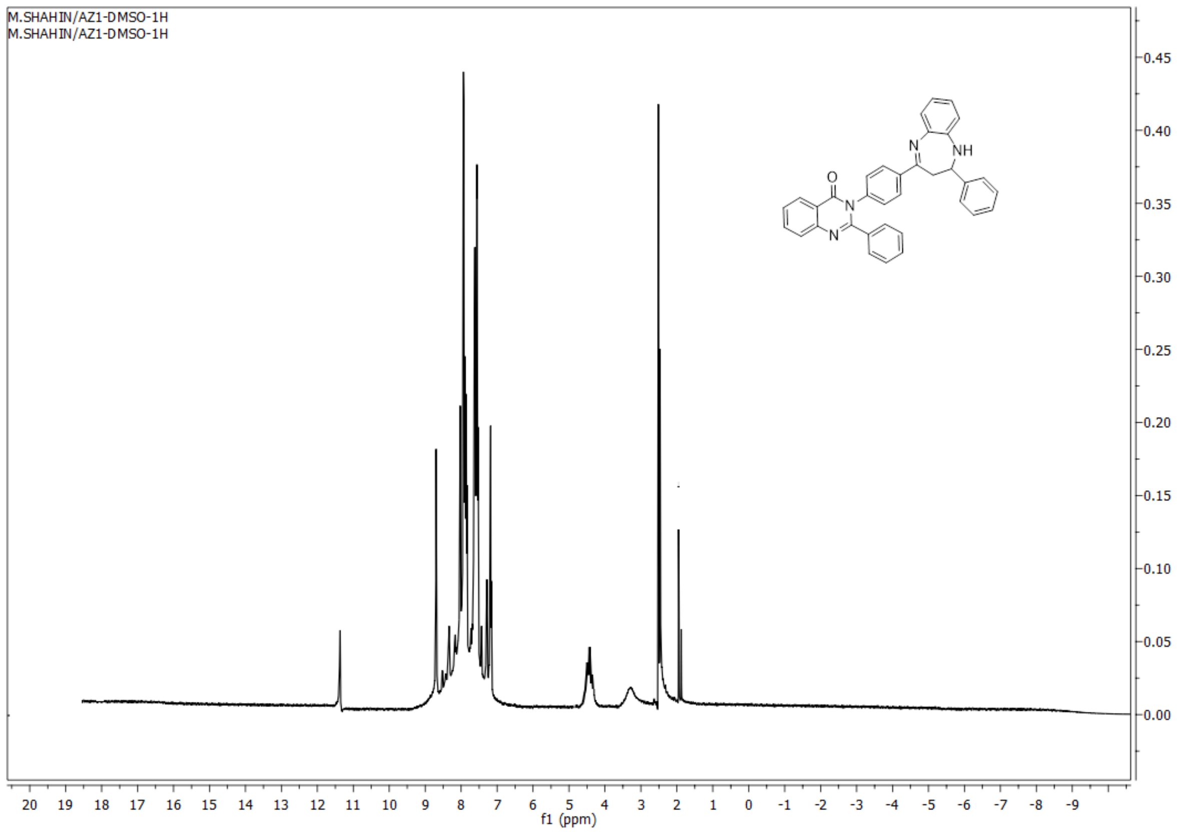

Figure S5: ^{1}H-NMR of compound 3c

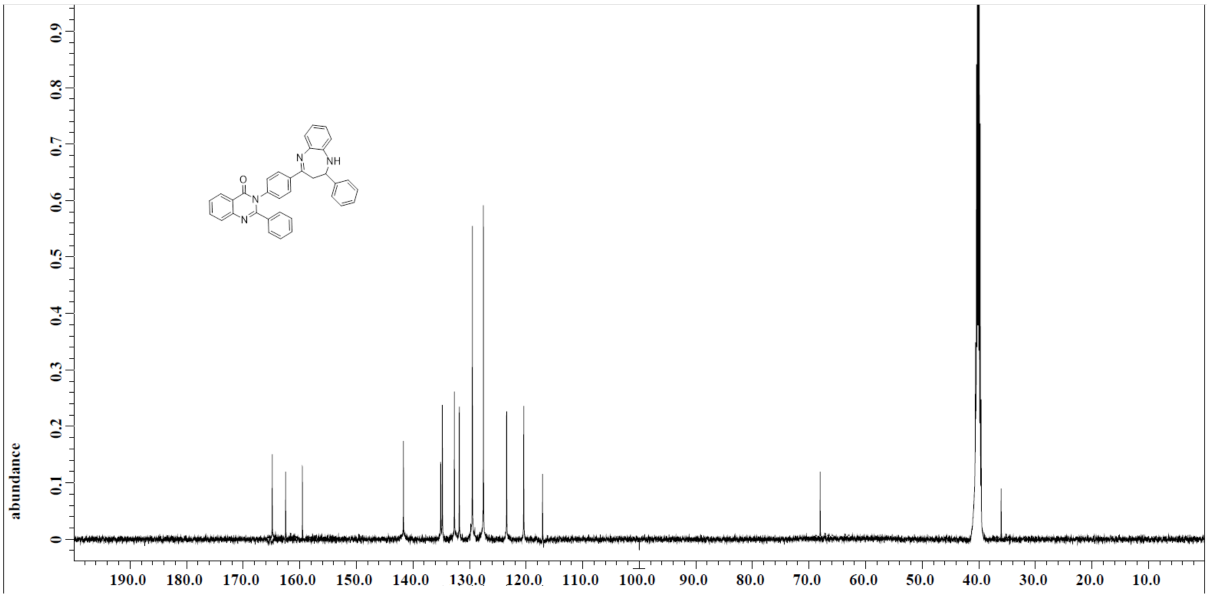

Figure S6: ^{13}C-NMR of compound 3c

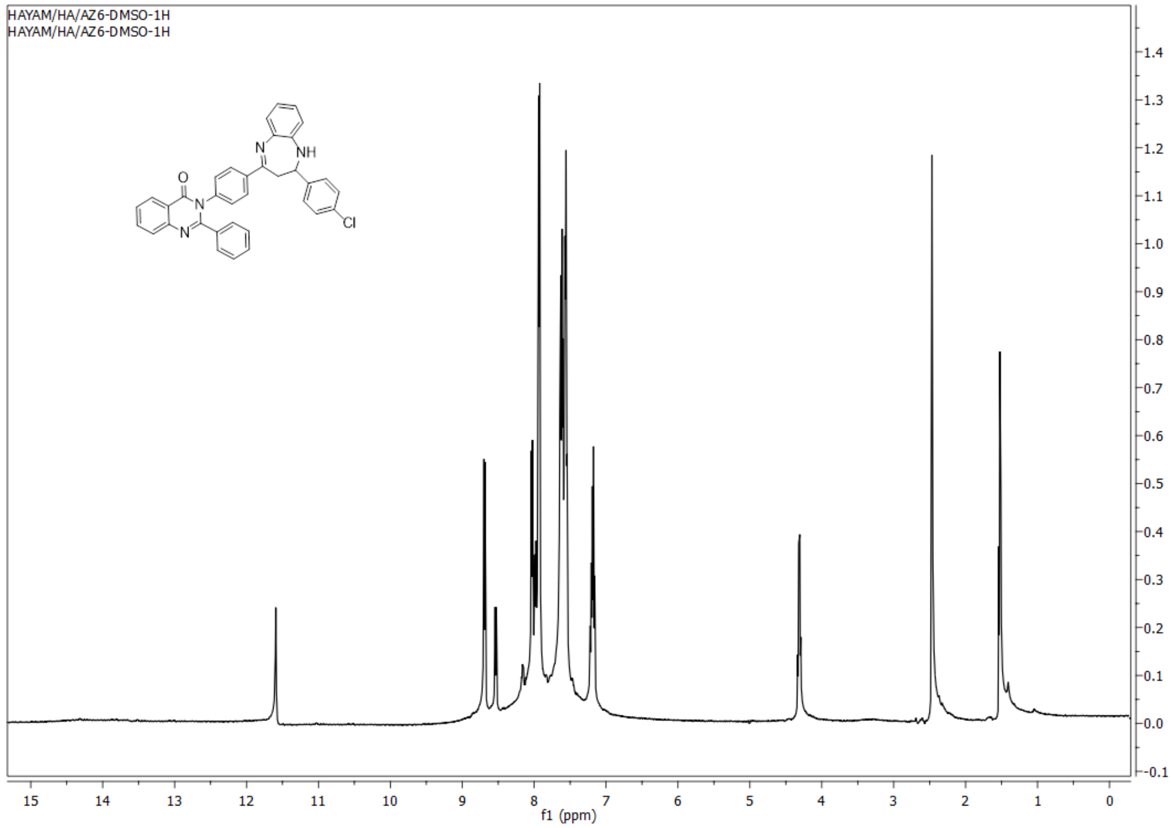

Figure S7: ^{1}H-NMR of compound 3d

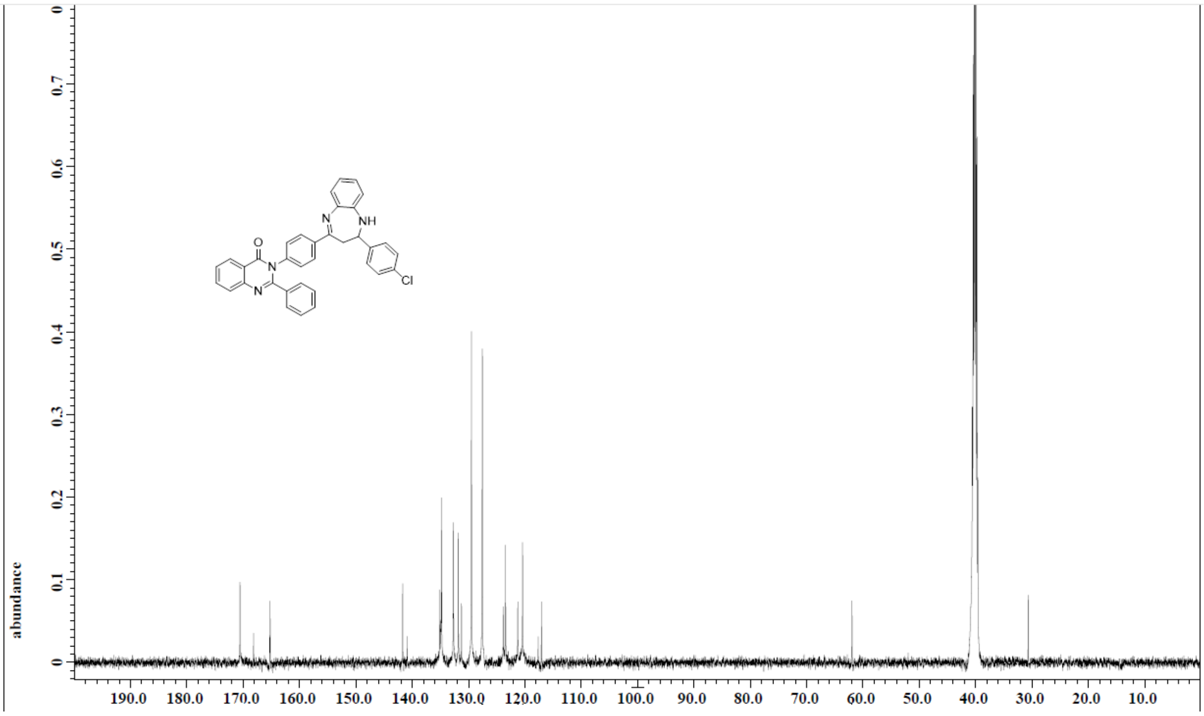

Figure S8: ^{13}C-NMR of compound 3d

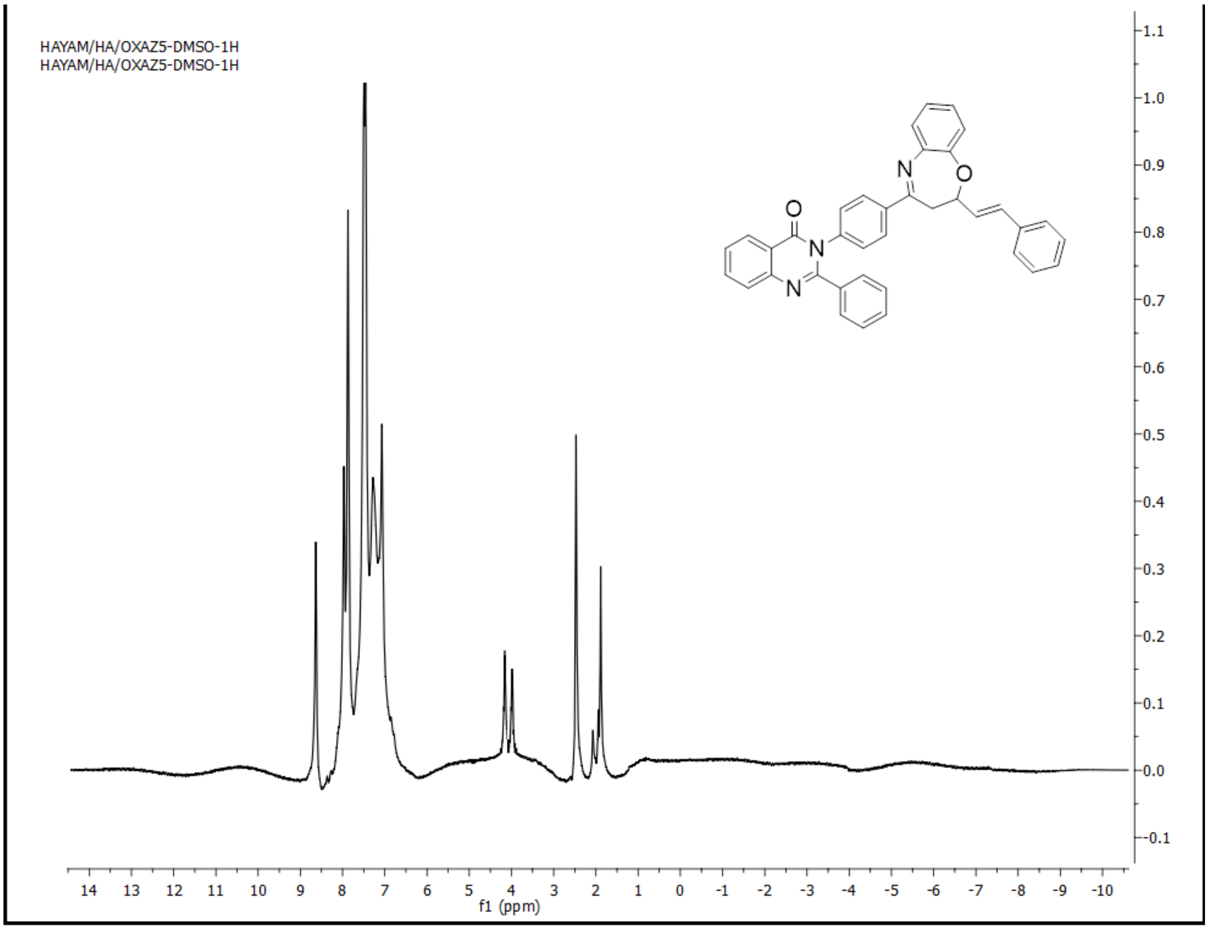

Figure S9: ^{1}H-NMR of compound 4a

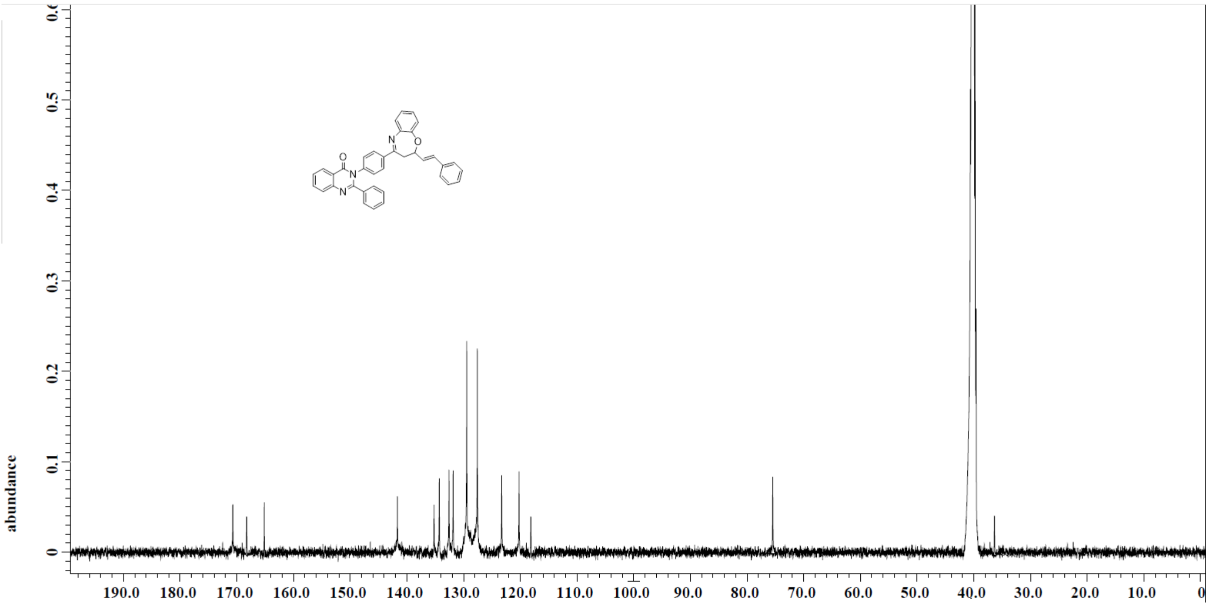

Figure S10: ^{13}C-NMR of compound 4a

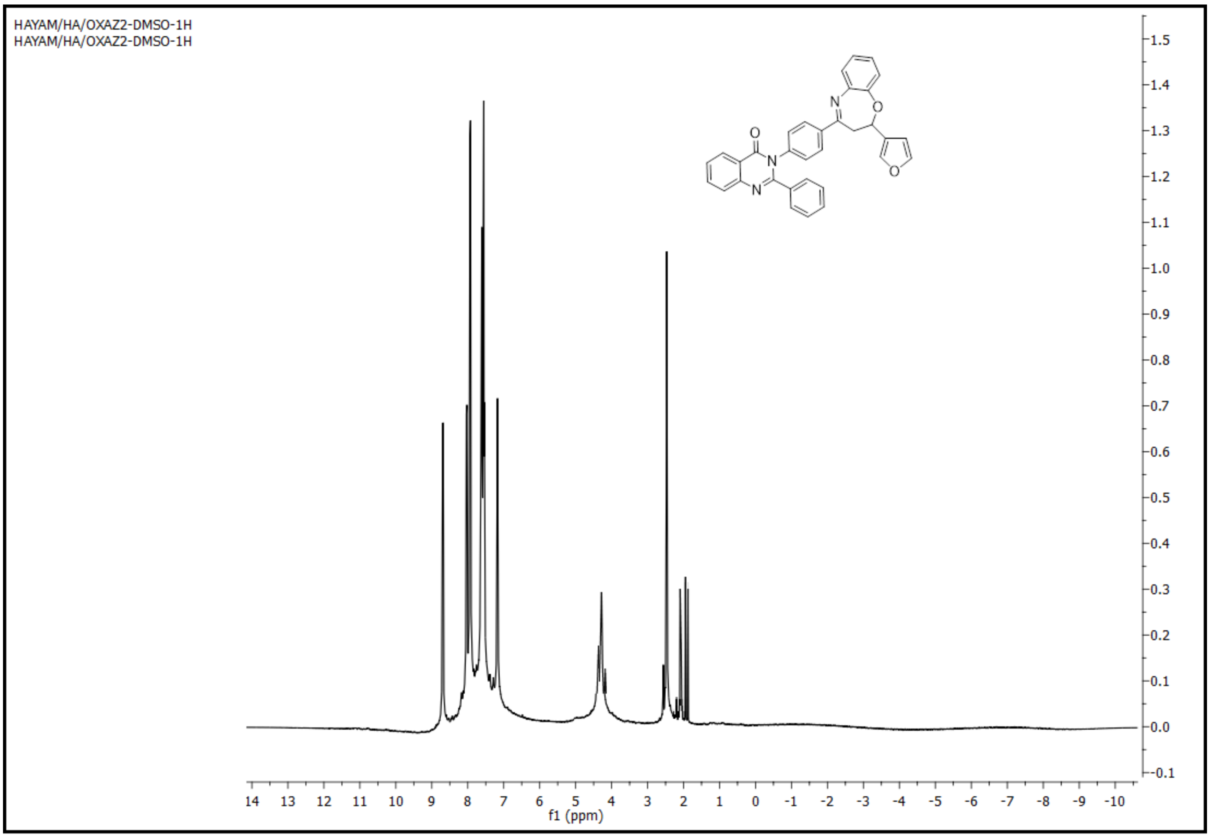

Figure S11: ^{1}H-NMR of compound 4b

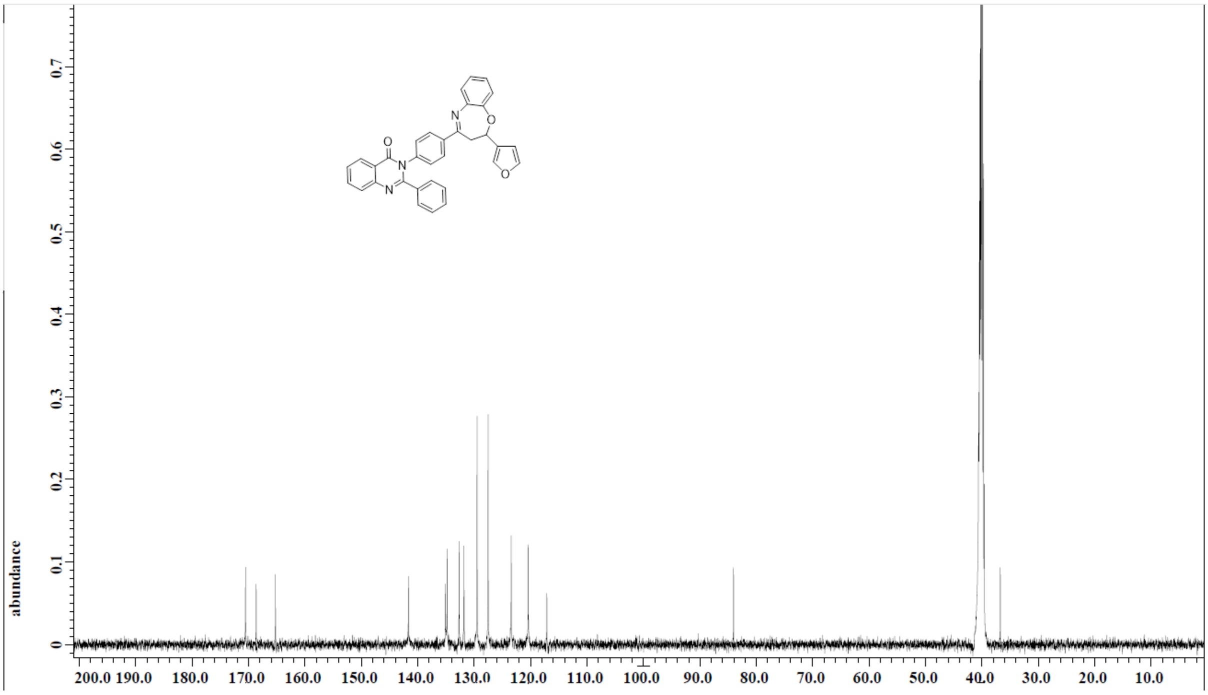

Figure S12: ^{13}C-NMR of compound 4b

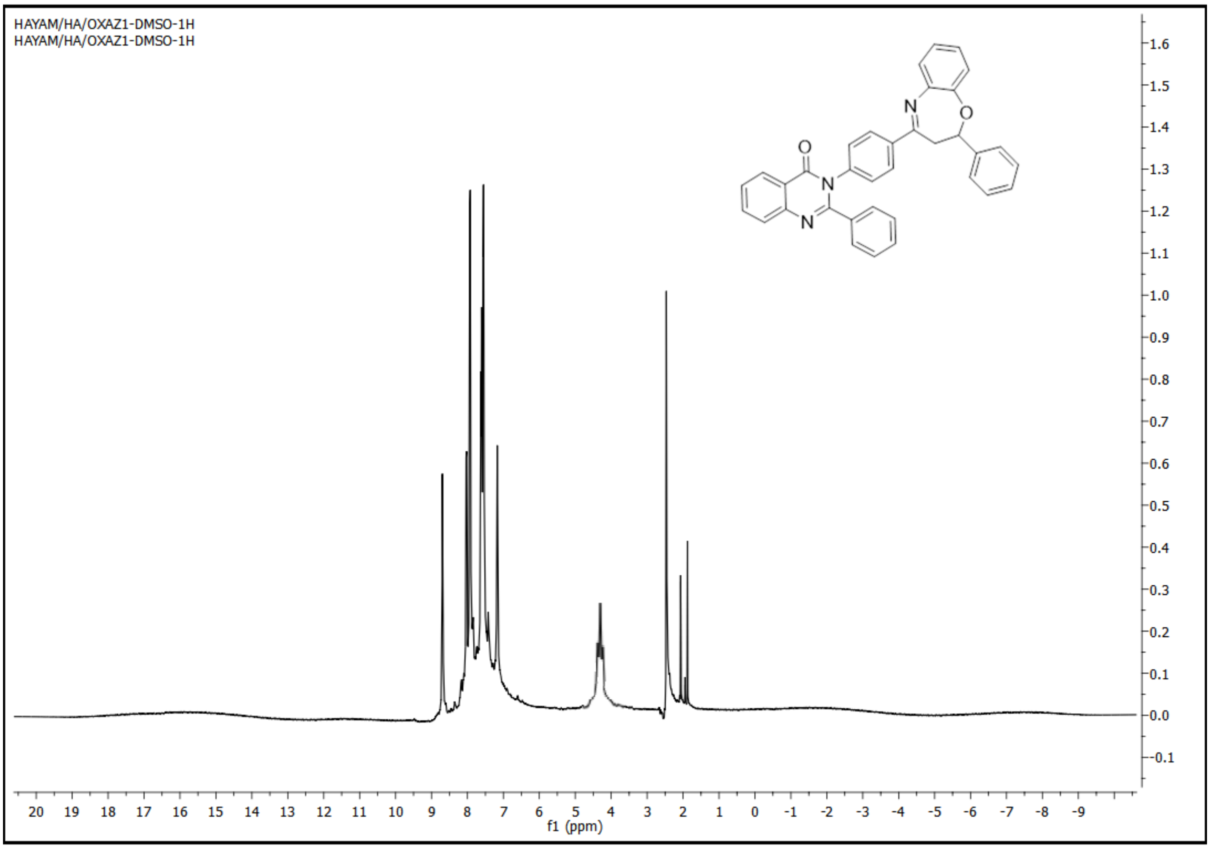

Figure S13: ^{1}H-NMR of compound 4c

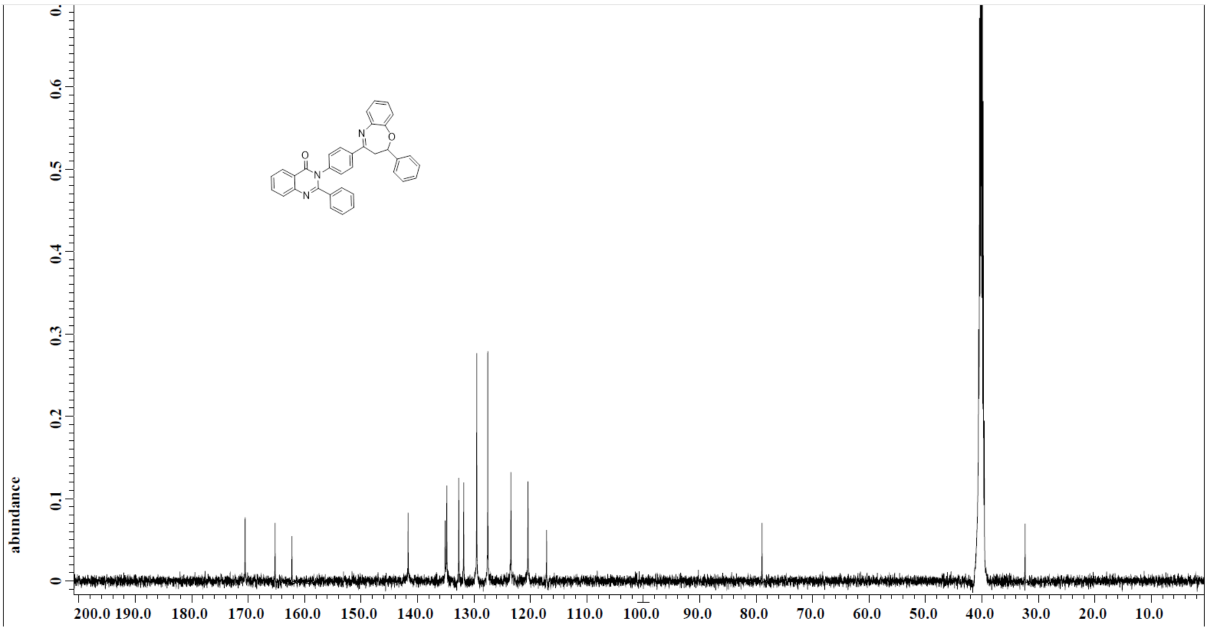

Figure S14: ^{13}C-NMR of compound 4c

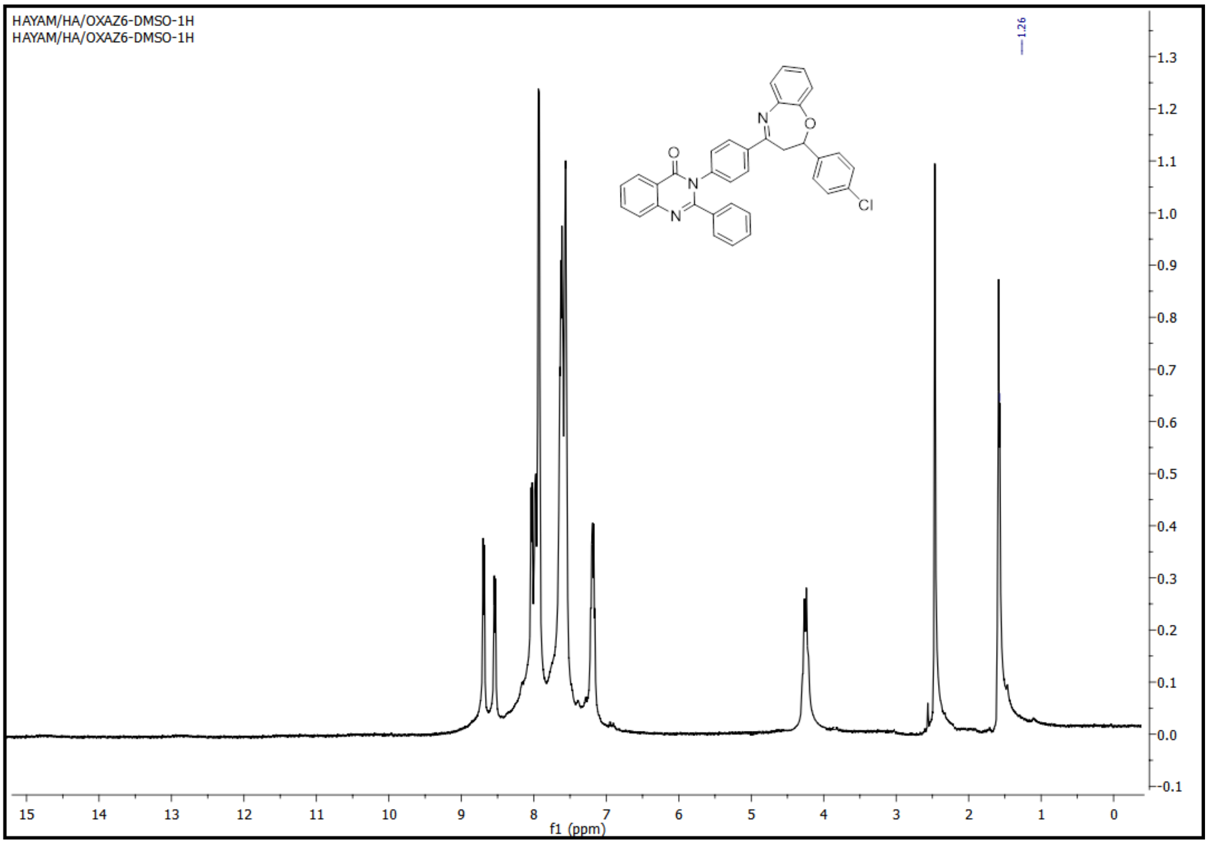

Figure S15: ^{1}H-NMR of compound 4d

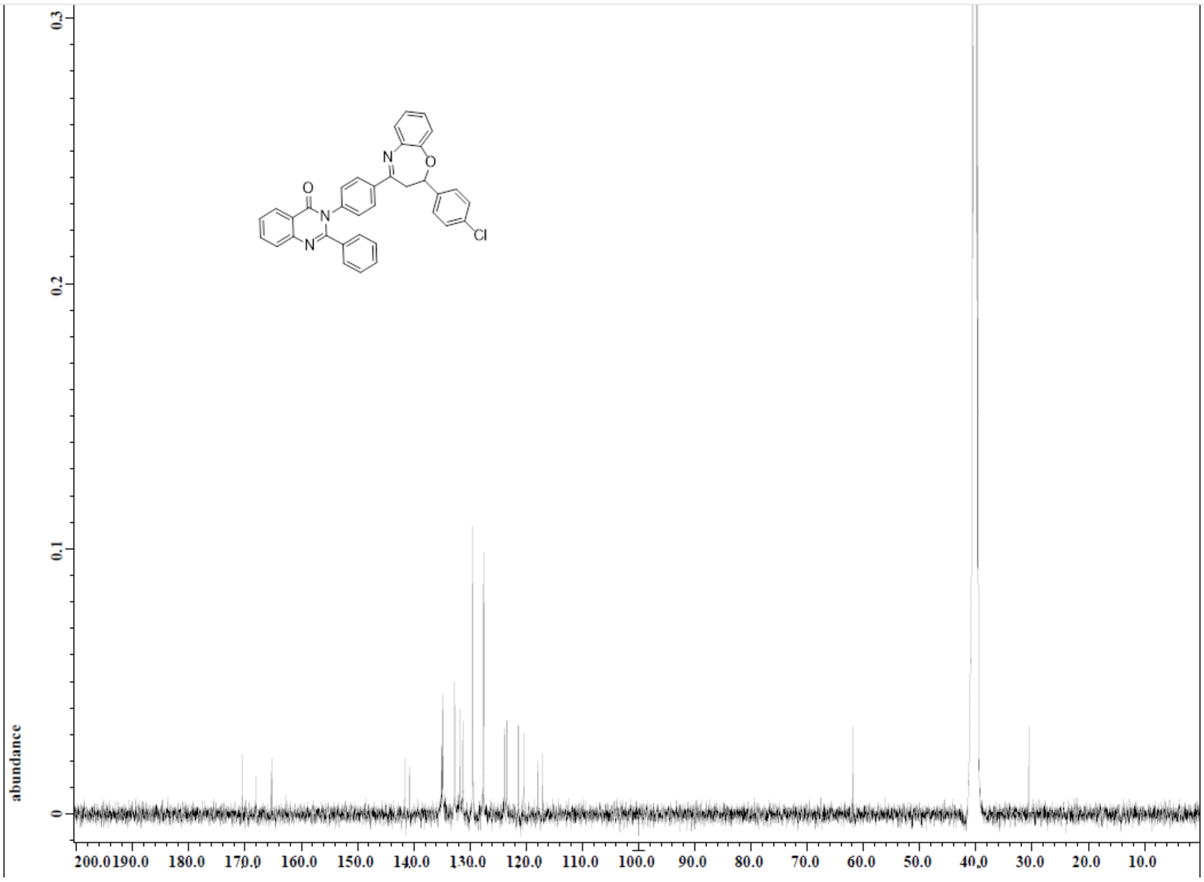

Figure S16: ^{13}C-NMR of compound 4d

Table S1. Calculated docking scores (kcal/mol) of all synthesized azepine derivatives and reference drugs with the target proteins.

Comps.	OMPA		Exo-1,3-beta-glucanase		SMO		SUFU/GLI-1	
	Docking Score (ΔG_{bind})	Docked complex (amino acid–ligand) interactions	Docking Score (ΔG_{bind})	Docked complex (amino acid–ligand) interactions	Docking Score (ΔG_{bind})	Docked complex (amino acid–ligand) interactions	Docking Score (ΔG_{bind})	Docked complex (amino acid–ligand) interactions
**3b**	-6.99	LEU401, THR392, ARG447, ARG405, LYS440, GLU444, THR355, ASN398, GLY393	-8.049	PHE144, PHE258, ARG309, ASN305, TYR317, ARG 312, PHE229, TYR317, ARG312, PHE229, TYR255, ASN146, GLU192	-7.44	ILEA465, PHEB457, ILEB454, TRPB371, PHEB455, LEUA516, TYRA233, ARGB451, PHEA237, ILEA520, PHEA523	-6.57	HISA164, GLUA551, TRPA163, GLNA375, PHEA456, LEUA458, GLYB127, SERB125, THRB128
**3c**	-6.79	PHE353, LYS536, LEU352, ARG405, ARG447, THR392, THR355	-7.51	PHE229, PHE144, PHE258, TYR255, PRO196, ASN146, LEU304	-7.07	ASPA229, LYSB539, TYRA233, GLUB447, ARGB451, PHEB455, PHEA523, LEUB458, THRB534, TRPB537, PHEA237, SERA232	-6.17	PROA453, GLUA375, ASPA476, GLUA376, BISA164, GLUA454, PROA475
**3d**	-7.046	ALA420, ASP419, GLY421, ILE400, LYS397, LEU401, LYS394, GLY393, THR392, GLY356	-7.68	TYR255, ASN305, LEU304, ASN146, PHE144, PHE258, PHE229, LEU194	-7.18	THRB538, LYSB39, ILEB454, GLUB447, ASPA229, PHEA523, ARGB451, TYRA233, THRB534, SERA232, PHEA237, TRPB537, ALAA236	-6.47	THRB128, HISA164, THRA205, PROA453, GLUA454, GLNA375, ASPA476
**4b**	-7.13	LEU352, THR355, GLY356, THR392, GLY393, PHE353, ARG405, ARG447	-7.47	PHE144, PHE258, TYR153, ASN146, ARG312, TYR317, ASP318, ASN305	-7.41	PHEA237, GLUB447, ARGB51, ALAA236, TYRA233, TRPB537, THRB538, LEUB542, PHEB247	-6.34	GLNA375, GLNA175, THRA205, GLUA206, PROA453, HISA164, GLUA454

**4c**	-6.99	THR355, THR392, GLY393, ARG447, ARG405, LEU401, PHE353	-7.05	ASP145, PHE144, TYR153, PHE258, PHE229, TYR255	-7.25	GLUB447, ASPA229, SERA232, PHEA237, ARGB451, LEUB458, THRB534, PHEA523, TRPB537, THRB538, TYRA233	-6.62	HISA164, THRA205, GLUA206, GLNA175, PROA453, SERA452, GLUA454, GLUA455, GLNA375
**4d**	-6.67	THR392, ARG447, LYS440, GLU444, LYS349, LEU352, LYS361, THR355	-6.86	PHE229, TYR317, LEU304, PHE258, PHE144, ASN146, TRP363	-7.03	GLUB447, ARGB451, PHEB455, PHEA523, LEUB458, TRPB537, TYRA233, THRB538, PHEA237	-6.18	THRA205, GLUA206, PROA453, GLUA454, GLUA375, THRB128, HISA164
**Ciprofloxacin (Reference antibiotic)**	-6.95	ARG447, THR392, ARG405, THR355, LYS440	-----	--------	-------	-------	-------	-------
**Clotrimazole (Reference antifungal)**	-----	----------	-6.23	PHE258, TYR317, PHE144, TYR153, LEU194, ARG309, ASN305, ASP318	-------	-------	--------	-------
**GANT-61 (Reference HH-GLI)**	-----	---------	------	---------	-7.62	THRB538, TYRA233, ILEB454, PHEA523, THRB534, ARGB451, PHEA237, TRPB537, GLUB447, LEUB458	-6.95	PROA453, THRA205, GLUA454, GLUA455, GLNA375, HISA164, THRB128

Chemicals and instrumentation

All analytical grade reagents were acquired from Sigma-Aldrich and utilized without additional purification unless otherwise stated. On Gallenkamp melting point equipment, all melting points were measured without modifications. Using the KBr disc approach, the Fourier transform infrared spectroscopy (FTIR) spectra were recorded on a Perkin-Elmer FTIR 1430 spectrophotometer. The ^{1}H nuclear magnetic resonance (NMR) spectra were recorded at 25°C in DMSO-d6 with TMS as an internal standard on spectrometer (400 MHz), and chemical shifts were reported in parts per million as δ values; ^{13}C NMR was set at 101 MHz, elemental studies for C, H, and N were also performed, and the results were found to be within 0.4 percent of theoretical values unless otherwise stated. Thin layer chromatography was used to track the reaction's progress
